# Dynamic changes in Lamin B1 and heterochromatin coincide with chromatin condensation during human erythropoiesis

**DOI:** 10.1186/s13059-026-04025-x

**Published:** 2026-03-24

**Authors:** Rui Wen, Qiqi Zhang, Yiqi Li, Yijin Chen, Yingru Jiang, Yingjie Lu, Junzhe Zhu, Delin Kong, Yulin Xu, Xinhua Liu, Pengxu Qian, Meng Zhang, He Huang, Dawei Huo

**Affiliations:** 1 Bone Marrow Transplantation Center of The First Affiliated Hospital & Liangzhu Laboratory, Zhejiang University School of Medicine, Hangzhou, China; 2 Institute of Hematology, Zhejiang University, Hangzhou, China; 3 Zhejiang Province Engineering Research Center for Stem Cell and Immunity Therapy, Hangzhou, China; 4 Department of Biochemistry and Molecular Biology, School of Basic Medicine Sciences, Hangzhou Normal University, Hangzhou, 311121 Zhejiang China

**Keywords:** Erythropoiesis, Chromatin structure, H3K9me3, Lamin B1, Heterochromatin

## Abstract

**Background:**

Chromatin condensation, accompanied by pronounced nuclear shrinkage, is a pivotal step in terminal erythroid differentiation and is essential for enucleation. However, the precise timing and molecular mechanisms initiating this process remain poorly understood, particularly as global transcriptional repression occurs only at the orthochromatic erythroblast stage.

**Results:**

Here we perform a comprehensive analysis of three-dimensional chromatin architecture dynamics during erythropoiesis using integrative epigenomic and imaging approaches. We demonstrate that chromatin condensation begins earlier than previously recognized, initiating at the late basophilic erythroblast stage. Mechanistically, these intergenic regions were tethered to the nuclear lamina (predominantly heterochromatic) and enriched in H3K9me3, which drive large-scale chromatin compaction. Furthermore, we find that the redistribution of H3K9me3 and dynamic remodeling of Lamin B1 are critical for this structural transition, directly impacting erythroid maturation.

**Conclusions:**

These findings reveal a previously unrecognized regulatory axis linking H3K9me3 and chromatin-lamina interactions, providing novel insights into the spatial and temporal control of chromatin organization during erythroid development.

**Supplementary Information:**

The online version contains supplementary material available at 10.1186/s13059-026-04025-x.

## Background

Erythropoiesis progresses from hematopoietic stem cells through a series of stages, including burst-forming unit-erythroid (BFU-E), colony-forming unit-erythroid (CFU-E), proerythroblast (ProE), basophilic erythroblast (Baso), polychromatic erythroblast (Poly), orthochromatic erythroblast (Ortho), reticulocyte, and finally mature red blood cells (RBCs). During this process, significant events occur, including cell size reduction, nuclear shrinkage, cell cycle exit, and enucleation. Chromatin condensation is considered a crucial preparation for the subsequent enucleation process [1–3]. Recent studies have used 3D genomics to investigate the chromatin condensation dynamics during erythroid differentiation. *GATA1* and *KLF1*, key erythroid-specific transcription factors, are essential not only for transcriptional regulation but also for the conversion and stability of topologically associating domains (TADs). From ProE to Ortho, long-range interactions are closely linked with heterochromatin [4–6]. Despite these insights, the dynamic progression of chromatin condensation, particularly the precise timing of its initiation and the underlying regulatory mechanisms, remains poorly understood. While several repressive regulators, including *HDAC5* [7], Polycomb complexes [8–10], *HP1* proteins [11], and *SET8* [12–14], have been implicated in erythroid development and chromatin regulation, their specific roles in chromatin condensation and the underlying molecular mechanisms have yet to be fully defined.

Lamin A and Lamin C (type A lamins), together with Lamin B1 and Lamin B2 (type B lamins), are key structural components of the nuclear lamina that have been implicated in the regulation of chromatin organization across a wide range of species [15–18]. A major function of the nuclear lamina is to anchor specific chromatin regions known as lamina-associated domains (LADs), which are typically gene-poor, enriched in repressive histone marks, and spatially positioned at the nuclear periphery. These heterochromatic domains play essential roles in maintaining higher-order genome architecture and contribute to the control of gene expression programs during the cell cycle, lineage specification, and terminal differentiation [19]. Knockout of Lamin B1 enhances heterochromatin de-compaction and increases chromatin mobility within the nucleus, suggesting its critical role in maintaining chromatin organization and nuclear stability [20]. In addition to regulating genome architecture, nuclear lamina proteins influence key mechanical properties of the cell, including stiffness, rigidity, and contractility, thereby linking nuclear structure with cellular biomechanics [20]. Nuclear lamina proteins undergo marked dynamic changes during erythroid differentiation. In particular, B-type lamins are subject to caspase-3-mediated proteolytic cleavage and WDR26-dependent ubiquitin-mediated degradation [21, 22]. These changes are accompanied by a significant increase in the Lamin A:B ratio and nuclear shrinkage during the late stages of differentiation [23]. In fibroblasts, Lamin B1 depletion increases nuclear sphericity and reduces cell volume [24], while in neutrophils, Lamin B1 plays a key role in regulating nuclear deformability. Such lamina remodeling is likely essential for the morphological transformations required for enucleation. Importantly, Lamin B1 is not uniformly lost from the nuclear periphery but undergoes spatially regulated depletion, leading to the formation of discrete nuclear openings [21]. These openings are thought to facilitate not only nuclear protein export but also large-scale reorganization of LADs and peripheral heterochromatin.

Here, we performed a comprehensive 3D genomics analysis of erythroid cells across six distinct stages of differentiation. Our results revealed a progressive increase in long-range chromatin interactions predominantly mediated by the B compartment, accompanied by pronounced chromatin compaction initiating at the late basophilic (LB) erythroblast stage. While TAD boundaries were globally disrupted during this process, TADs specifically associated with erythropoiesis remained comparatively stable, suggesting selective preservation of functionally relevant chromatin domains. The B compartment showed marked enrichment of heterochromatic histone modifications, particularly H3K9me3, which correlated strongly with the extent of chromatin condensation. These structural changes were most evident within LADs, where a significant reorganization of H3K9me3 deposition occurred. Importantly, dynamic alterations in Lamin B1 localization closely mirrored these chromatin remodeling events, with a depletion of Lamin B1 at the nuclear periphery coinciding temporally with the LB stage. Functional assays demonstrated that stage-specific Lamin B1 depletion exerted distinct effects on erythroid differentiation, underscoring its critical and context dependent regulatory role. Collectively, our findings emphasize the pivotal contribution of H3K9me3-mediated heterochromatin interactions and Lamin B1 dynamics in orchestrating the spatiotemporal reorganization of chromatin architecture throughout erythropoiesis.

## Results

### Long range interactions initiate at the late basophilic erythroblast stage

We developed an efficient method for isolating primary human erythroblasts derived from cord blood CD34 + cells at distinct terminal differentiation stages across a 15–20 days process, encompassing four distinct culture phases (Additional file 1: Fig. S1A). Cells were classified into distinct stages based on their surface marker expression. At day 7, CD36^−^GPA^−^ cells were defined as BFU, CD36^+^GPA^−^ cells as CFU, and CD36^+^GPA^+^ cells as ProE. At day 13, GPA^high^ CD105^high^ cells were defined as EB (early Baso) and GPA^high^ CD105^dim^ cells as LB (late Baso). At day 15, GPA^high^ CD105^low^ cells were defined as Poly and GPA^high^ CD105^−^ cells as Ortho [25]. Cells at each developmental stage were isolated by flow cytometry, and their morphology was confirmed using Giemsa staining (Additional file 1: Fig. S1B, C).

To investigate the 3D chromatin architecture during erythroid differentiation, we applied a modified Bridge Linker Hi-C protocol [26] to erythroblasts isolated at the defined developmental stages described above. For each stage, we generated high-coverage chromatin interaction maps, obtaining at least 0.5 billion uniquely mapped paired-end reads per sample (Additional file 2: Table S1), ensuring sufficient depth for robust downstream analyses. Biological replicates exhibited high reproducibility, as demonstrated by strong correlation coefficients between datasets (Additional file 1: Fig. S1D), confirming the reliability of our Hi-C data. This comprehensive dataset enabled detailed characterization of chromatin structural dynamics across erythroid maturation.

**Fig. 1:**
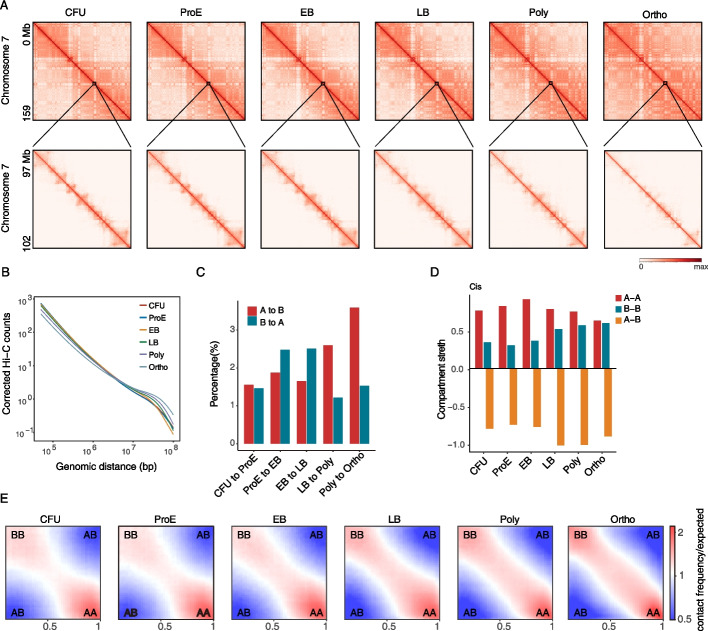
Stage-specific emergence of long-range chromatin interactions during erythroid differentiation, initiated at the LB stage. **A** Heatmaps showing normalized Hi-C interaction frequencies (200-kb bin) of chromosome 7 at different stages of erythroid differentiation in vitro. Zoomed-in views (40-Kb bin) are also shown. **B** Corrected Hi-C counts across all genome for each stage of differentiation are shown relative to the genomic distance. **C** Changes of A/B Compartment status between different stages of erythroid differentiation. **D** Log₂ ratios of observed versus expected Hi-C contact intensities were calculated for A–A, B–B, and A–B compartment interactions at each stage of erythroid differentiation. **E** Saddle plots showing the change in compartment interactions at each stage of erythroid differentiation

Further analysis of chromatin interactions during erythroid differentiation revealed a significant increase in long-range interactions spanning over 50 Mb, accompanied by a gradual decline in short-range contacts under 50 Mb (Fig. 1A, B). Interchromosomal interactions also showed pronounced enhancement, with this reorganization initiating at the LB stage (Additional file 1: Fig. S2A). To characterize genome compartmentalization dynamics, we employed principal component analysis (PCA) using PC1 values, which identified approximately 2% of the genome undergoing compartment switching throughout differentiation. This switching was predominantly characterized by a unidirectional shift from transcriptionally active A compartments toward repressive B compartments, with transitions from A to B occurring more frequently than the reverse (Fig. 1C). Correspondingly, intra-compartment interactions exhibited complementary trends: A–A contacts appear relatively stable across differentiation, whereas B–B interactions strengthened, reflecting distinct spatiotemporal dynamics of euchromatin and heterochromatin during differentiation (Fig. 1D, E). Interchromosomal interactions displayed a distinct pattern, with both A–A and B–B contacts diminishing while A–B interactions increasing (Additional file 1: Fig. S2B-D), suggesting enhanced cross-compartment communication. We analyzed the genes located within the A/B compartment switching regions throughout the differentiation process (Additional file 1: Fig. S2E, F). The results show that regions switching to A in later stages already exhibited certain transcriptional activity early on and maintained their expression to some extent. In contrast, regions transitioning from A to B displayed a more pronounced shift from an active to a silenced transcriptional state. Collectively, these findings highlight that chromatin condensation during erythroid maturation involves enhanced B-B compartment interactions and structural reprogramming beginning at the LB stage.

### The global reprogramming of TADs during erythropoiesis

**Fig. 2:**
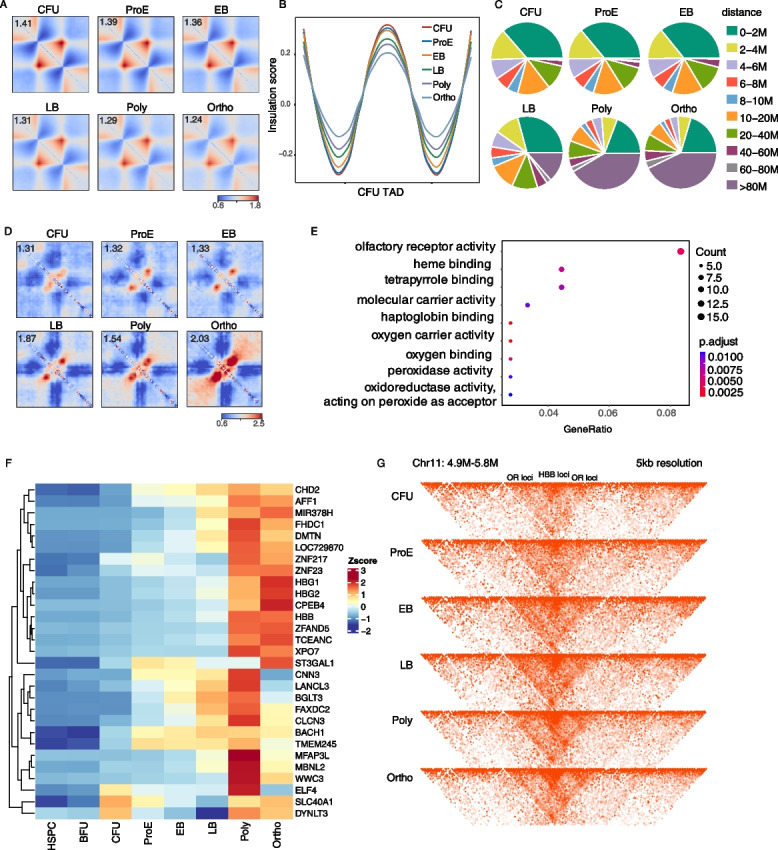
The global reprogramming of TADs during erythropoiesis. **A** Heatmaps showing the normalized average interaction frequencies for TADs and the neighboring ± 0.5 Mb TAD regions at each stage of erythroid differentiation. **B** The average insulation scores at TADs (defined in CFU) and nearby regions are shown. **C** Relative proportion was calculated from the amount of inter-TAD interactions separated by genomic distances within a certain window divided by the amount of total interactions. **D** Heatmaps showing the normalized average interaction frequencies of TADs that exhibited increased stability at later stages of erythroid differentiation. **E** Results of GO enrichment analysis of upregulated genes overlapping TADs that exhibited increased stability at later stages of erythroid differentiation. **F** Heatmaps showing the expression dynamics of upregulated genes overlapping TADs that exhibited increased stability during erythroid differentiation. Values were normalized using z-scores. **G** The structural differences of HBB clusters for each stage of differentiation. The heatmap is displayed at 5 kb resolution in washU

Although TADs are generally stable across cell types and conserved among species, significant reorganization of TAD architecture occurs during erythropoiesis. However, it remains unclear whether such chromatin remodeling initiates at the LB stage. To this end, we quantified the relative variance in TAD strength across all differentiation stages. Our analysis revealed that TAD insulation and boundary strength remained largely stable during the initial three stages of differentiation but began to decline markedly starting at the LB erythroblast stage (Fig. 2A, B; Additional file 1: Fig. S2G). This reduction in boundary insulation correlated temporally with the onset of extensive transcriptional changes, consistent with previous reports describing widespread silencing of non-essential erythroid genes and progressive loss of chromatin accessibility during terminal maturation [27]. Furthermore, we examined the frequency of chromatin interactions between TADs across varying genomic distances, which aligned well with contact matrix observations. There was a pronounced increase in ultra-long-range interactions spanning distances greater than 80 Mb (Fig. 2C), suggesting a genome-wide remodeling of higher-order chromatin contacts coinciding with TAD weakening and chromatin compaction.

Despite the general weakening of TAD structures during erythroid differentiation, we identified a subset of TADs exhibiting increased structural strength (Fig. 2D). Interestingly, these reinforced domains were significantly enriched for genomic regions harboring genes essential for terminal erythropoiesis (Fig. 2E). As shown in the heatmap, several of these genes demonstrated stage-specific upregulation in the later phases of differentiation. Several key regulators functionally have been validated key regulators, such as *XPO7*, which plays a critical role in nuclear protein export [28], and *CPEB4*, a well-characterized modulator of erythroid development [29]. Other genes residing within these strengthened TADs represent promising candidates for future functional studies (Fig. 2F). Functional enrichment analysis of genes located within these domains revealed significant associations with processes central to erythroid function, including heme binding, tetrapyrrole binding, oxygen carrier activity, and notably the HBB locus. Although the HBB locus lies adjacent to olfactory receptor (OR) genes, which contribute to an apparent enrichment of “olfactory receptor activity” in Gene Ontology (GO) analysis, the neighboring gene set predominantly pertains to hemoglobin metabolism and red blood cell function. These pathways align closely with the specialized physiological roles of mature erythroblasts (Fig. 2G). Conclusively, these findings suggest that during the late stages of erythroid maturation, despite the general weakening of TAD structures during erythroid differentiation, critical erythropoiesis genes still maintain robust TAD organization to sustain their transcriptional activity.

### H3K9me3 enrichment coincides with long-range interaction patterns in the B compartment

**Fig. 3:**
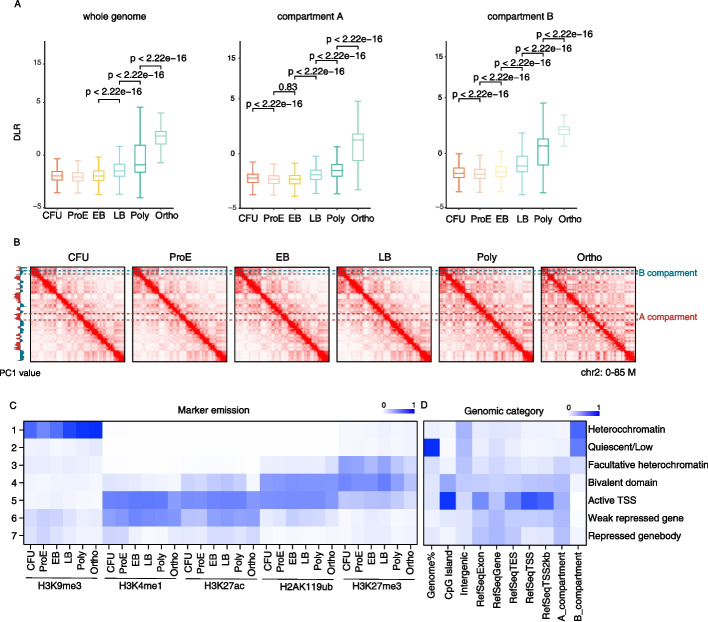
H3K9me3 enrichment coincides with long-Range interaction patterns in the B compartment. **A** Changes in DLR (Distal-to-Local [log2] Ratio), with erythroid differentiation in different regions of the chromosome (whole genome(left), compartment A(middle), compartment B(right)). The *p*-value is also shown. **B** Contact matrices of the 0–80 M region of chromosome 2, at different stages of erythroblasts differentiation. The different partitions of Compartment A/B are also labelled on the left (A labelled in red, B labelled in green). **C** Genome-wide chromatin states were defined by integrating histone modification ChIP-seq data across erythroid differentiation stages. Genomic regions were grouped into 7 clusters based on their epigenetic signatures using ChromHMM, color intensity indicates the signal strength of each histone mark in each cluster and stage. The corresponding definitions are shown on the right side of the figure. **D** The chromatin-state functional enrichment map shows the relative genomic coverage of each chromatin state. Chromatin states were derived from ChromHMM analysis and categorized based on their association with CpG islands, intergenic, exon, refseq genes, refseq TES, refseq TSS, A/B compartment. Enrichment was calculated relative to the overall genome distribution

To further elucidate the mechanisms underlying chromatin compaction during erythroid differentiation, we performed DLR (Distal-to-Local Ratio) analysis across the entire genome, as well as stratified by A and B compartments. DLR is defined as the log₂ ratio of distal Hi-C interactions (greater than 3 megabases [Mb]) to local Hi-C interactions (within 3 Mb) on the same chromosome. Our results revealed that the B compartment exhibited significantly higher DLR values than the A compartment, with a marked increase starting at the LB stage (Fig. 3A). Together, A representative genomic region on chromosome 2 (0–85 Mb) vividly illustrated this dynamic structural reorganization (Fig. 3B). To further assess changes in interchromosomal interaction patterns across differentiation stages, we calculated the ICF (Interchromosomal Interaction Frequency) score. However, we observed no significant differences in ICF scores among the analyzed genomic compartments (Additional file 1: Fig. S3A). Collectively, these findings suggest that the large-scale reorganization of chromatin architecture during terminal erythropoiesis is predominantly driven by enhanced long-range interactions within the B compartment.

Given the critical role of histone modifications in regulating chromatin architecture, we performed ChIP-seq profiling of five key histone marks—H3K9me3, H3K27me3, H3K27ac, H2AK119ub, and H3K4me1, across all major stages of erythroid differentiation. These marks were selected to capture both repressive (H3K9me3, H3K27me3, H2AK119ub) and active (H3K27ac, H3K4me1) chromatin features. To systematically characterize the combinatorial patterns of these modifications and their dynamics during lineage progression, we applied ChromHMM to perform genome-wide chromatin state segmentation. This unsupervised learning approach enabled us to define distinct chromatin states, annotate functional genomic elements, and track state transitions throughout erythropoiesis. The resulting chromatin state maps provided a high resolution framework to link epigenomic changes with large-scale chromatin reorganization and gene regulatory programs during terminal differentiation.

Our results revealed that the B compartment was predominantly enriched for H3K9me3 (Cluster 1), with a progressive increase observed during differentiation (Fig. 3C). This cluster was largely localized to intergenic regions (Fig. 3D). In contrast, other histone modifications were primarily associated with the A compartment (Clusters 3–7) (Fig. 3C). Two Polycomb-associated repressive marks exhibited divergent genomic distributions: H3K27me3 was preferentially enriched in Clusters 3 and 4, which displayed relatively low levels of H3K4me1 and H3K27ac, whereas H2AK119ub was preferentially enriched in Cluster 4、5, marked by high H3K27ac signals (Fig. 3C, D; Additional file 1: Fig. S3B). Together, by integrating these epigenomic profiles with Hi-C data, our findings indicate that, although multiple repressive histone modifications are linked to chromatin compaction, H3K9me3-mediated heterochromatin formation shows the strongest correlation with B compartment during erythroid differentiation.

### H3K9me3 exhibits progressive redistribution during terminal erythroid differentiation

**Fig. 4:**
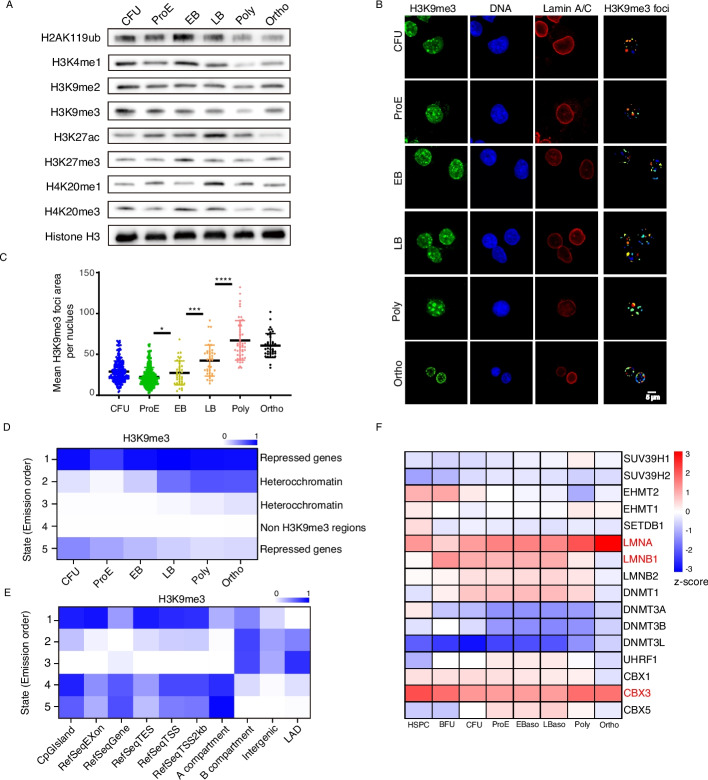
H3K9me3 exhibits progressive redistribution during terminal erythroid differentiation. **A** Western blot analysis of different histone markers levels at different erythroid differentiation stages. **B** Immunofluorescence analysis of H3K9me3 distribution during erythroid differentiation. Representative images show changes in H3K9me3 foci localization across differentiation stages. Foci detection and signal quantification were performed using CellProfiler. The scale bar represents 5 μm. **C** Scatter plots represent the area size of individual H3K9me3 foci per nucleus across different stages of erythroid differentiation. **D** Heatmap showing genome-wide dynamics of H3K9me3-based chromatin states across erythroid differentiation stages. Genomic regions were segmented into five distinct chromatin states using ChromHMM, based on their H3K9me3 signal intensity profiles, the color intensity in each cluster reflects the signal strength. The corresponding definitions are shown on the right side of the figure. **E** The chromatin-state functional enrichment map shows the relative genomic coverage of each chromatin state. Chromatin states were derived from ChromHMM analysis and categorized based on their association with CpG islands, intergenic, exon, refseq genes, refseq TES, refseq TSS, A/B compartment, LAD Enrichment was calculated relative to the overall genome distribution. **F** Heatmap showing the changes in mRNA expression levels of key heterochromatinassociated genes across different stages with expression values normalized by z-score

To investigate how heterochromatin regulates chromatin compaction during erythroid differentiation, we performed more detailed experimental analyses of relevant histone modifications. At the global level, H3K9me3 levels at terminal differentiation stages were lower than those at the CFU and ProE stages but remained relatively stable thereafter. H4K20me1 showed a significant increase at the LB stage, whereas other heterochromatin-associated marks, including H3K9me2 and H4K20me3, exhibited no notable changes throughout the differentiation time course (Fig. 4A). In contrast, the active modification H3K27ac showed a marked decrease (Fig. 4A). We further carried out immunofluorescence staining to establish a link between histone modifications and DNA condensation. The results revealed a strong correlation between H3K9me3 and DNA condensation: during differentiation, H3K9me3 foci gradually increase in size and become localized at the nuclear periphery by the Ortho stage and coalesced with DNA condensation (Fig. 4B). Quantitative analysis of these foci, after enhancement and filtering, showed an increase in both size and number over time, culminating in peripheral localization at the nuclear lamina by the orthochromatic stage (Fig. 4B, C). In contrast, another heterochromatic mark, H3K9me2, did not show a clear spatial correlation with DNA condensation (Additional file 1: Fig. S4A). The Polycomb-associated repressive mark H3K27me3 was broadly distributed across the chromatin, but exhibited relatively lower enrichment at sites of internal DNA aggregation (Additional file 1: Fig. S4B). Surprisingly, H2AK119ub was excluded from condensed DNA regions specifically at the orthochromatic stage (Additional file 1: Fig. S4C). This observation aligns with previous studies reporting a dual regulatory function of H2AK119ub in gene expression [30]. These results suggest that chromatin compaction during erythroid differentiation is mediated by the folding of heterochromatin regions enriched with the H3K9me3 mark and that this process becomes increasingly associated with the nuclear lamina at later stages.

To identify the genomic regions undergoing such structural changes, we performed a genome-wide profiling analysis of H3K9me3 (Fig. 4D, E). Among the five identified clusters, cluster 4 was negative for H3K9me3 signal, whereas clusters 1 and 5 showed no significant change or even a gradual decrease in H3K9me3 levels during differentiation. These regions were predominantly located within the A compartment, which corresponds to gene-dense regions of the genome. In contrast, clusters 2 and 3 exhibited a progressive increase in H3K9me3 signals during differentiation and were primarily localized to the B compartment, which is largely composed of intergenic regions. These regions also overlapped significantly with LADs. This finding indicates that intergenic heterochromatin plays a central role in long-range interactions within the B compartment and is closely linked to LAD organization. A representative track plot further illustrates the dynamic changes in H3K9me3 across different clusters (Additional file 1: Fig. S4D). We further analyzed the expression dynamics of genes involved in chromatin organization during differentiation (Fig. 4F). Among them, *CBX3*, a gene implicated in mediating chromatin interactions, maintained high expression levels at later stages. Changes in the expression of nuclear laminas, *LMNA* and *LMNB1*, were consistent with previously reported protein-level observations [21–23]. Taken together, these findings suggest that the dynamic remodeling of intergenic heterochromatin is strongly correlated with chromatin compaction and may be associated with nuclear laminas.

### Dynamic regulation of Lamin B1 is closely associated with chromatin condensation

**Fig. 5:**
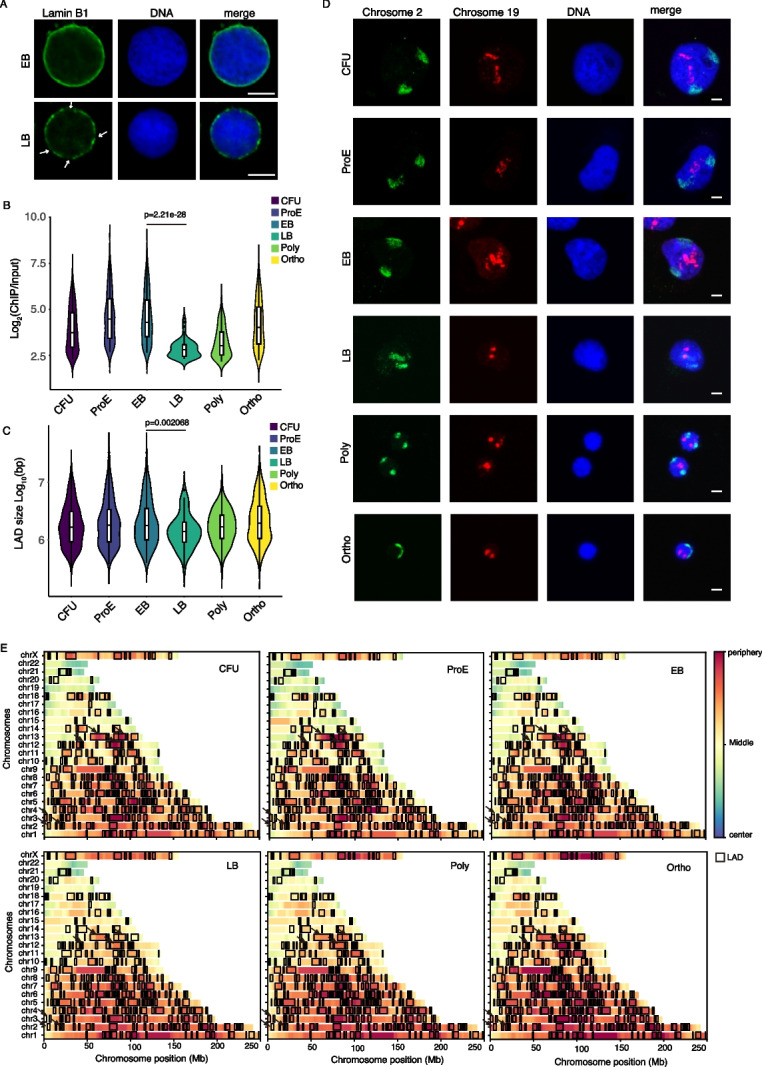
Dynamic regulation of Lamin B1 is closely associated with chromatin condensation. **A** Immunofluorescence showing the Lamin B1 protein expression in the nuclear membrane of EB and LB stages. The scale bar represents 5 μm. **B** Violin plots show the distribution of log2-transformed ChIP/Input enrichment levels of Lamin B1 within LAD regions at different erythroid differentiation stages. The *p* values were determined by two-tailed Student’s t-test. **C** Violin plots show the distribution of LAD sizes (in base pairs, log10-transformed) at different stages of erythroid differentiation. The *p* values were determined by two-tailed Student’s t-test. **D** Chromatin painting showing spatial repositioning of chromosome 2 (green) and chromosome 19 (red) during erythroid differentiation. The scale bar represents 5 μm. **E** Heatmaps showing average chromatin positioning across 400 simulated nuclear structures per erythroid differentiation stage. For each stage of erythropoiesis, 400 three-dimensional nucleus models were generated based on chromatin interaction data and structural constraints. The averaged spatial coordinates of genomic bins (1 Mb resolution) are displayed as heatmaps. Blue TADs represent regions consistently localized toward the nuclear center, while red TADs show stable association with the nuclear periphery. LAD regions are outlined in black, highlighting their preferential localization at the nuclear lamina

As previously described, Lamin B1 plays a crucial role in chromatin compaction. To further investigate this relationship, we performed immunofluorescence staining and observed localized gaps of Lamin B1, consistent with previous reports [21]. During the LB stage, Lamin B1 exhibited a marked loss from the nuclear lamina, and the regions of loss precisely colocalized with H3K9me3-enriched domains (Fig. 5A and Additional file 1: Fig. S5A). This depletion is often associated with increased chromatin mobility [20], which aligns temporally with the onset of chromatin condensation. We also knocked down *LMNB1* expression during differentiation and examined the levels and localization of H3K9me3. The results showed that *LMNB1* knockdown led to a reduction in H3K9me3 levels. Moreover, immunofluorescence analysis revealed that loss of Lamin B1 dramatically altered H3K9me3 nuclear distribution: whereas control nuclei displayed distinct punctate H3K9me3 foci, H3K9me3 in knockdown cells became diffusely localized along the nuclear periphery and was virtually absent from the nuclear interior (Additional file 1: Fig. S5B, C). We also observed that *LMNB1* knockdown exerted certain effects on the cell cycle and cell viability (Additional file 1: Fig. S5D, E). Analysis of Lamin B1 ChIP-seq data revealed that both the intensity and size of LADs were significantly reduced at the LB stage (Fig. 5B, C). Local track views clearly illustrated these changes (Additional file 1: Fig. S6A). For B compartment in chromosome 2, which is predominantly composed of intergenic regions and LADs, we observed an increase in H3K9me3 signal and structural reorganization of LADs. In contrast, chromosome 19, whose B compartment mainly encompasses gene-rich regions lacking LAD structures, showed no significant change in H3K9me3 levels.

To further explore the spatial organization of these chromosomes, we conducted chromosome painting experiments (Fig. 5D). Chromosome 2 was predominantly localized at the nuclear periphery and exhibited a transient inward migration during the LB stage, followed by reattachment to the nuclear lamina accompanied by a noticeable reduction in volume. In contrast, chromosome 19, which lacks LAD structures, remained centrally located within the nucleus throughout differentiation.

Using Hi-C data, we reconstructed a comprehensive 3D model of chromatin organization across erythroid differentiation using Chrom3D (Additional file 1: Fig. S6B). The modeling encompassed all major differentiation stages and was carried out based on 400 simulated 3D structures, following established protocols from previous studies [31]. We calculated the average distance of genomic region per 1 Mb from the nuclear center. Consistent with previous observations, larger chromosomes tended to localize toward the nuclear periphery [31]. During terminal erythropoiesis, LAD-associated chromatin regions exhibited increased association with the nuclear lamina. However, we observed a marked detachment from the nuclear periphery at the LB stage, particularly evident in chromosomes 12 and 13. This dynamic re-localization reflects progressive heterochromatin compaction at the nuclear lamina, coinciding with changes in laminar organization during erythroid maturation (Fig. 5E). Overall, these data hint that dynamic alterations in Lamin B1 localization were closely correlated with chromatin remodeling events.

### Loss of Lamin B1 impairs heterochromatin establishment and cell differentiation

**Fig. 6:**
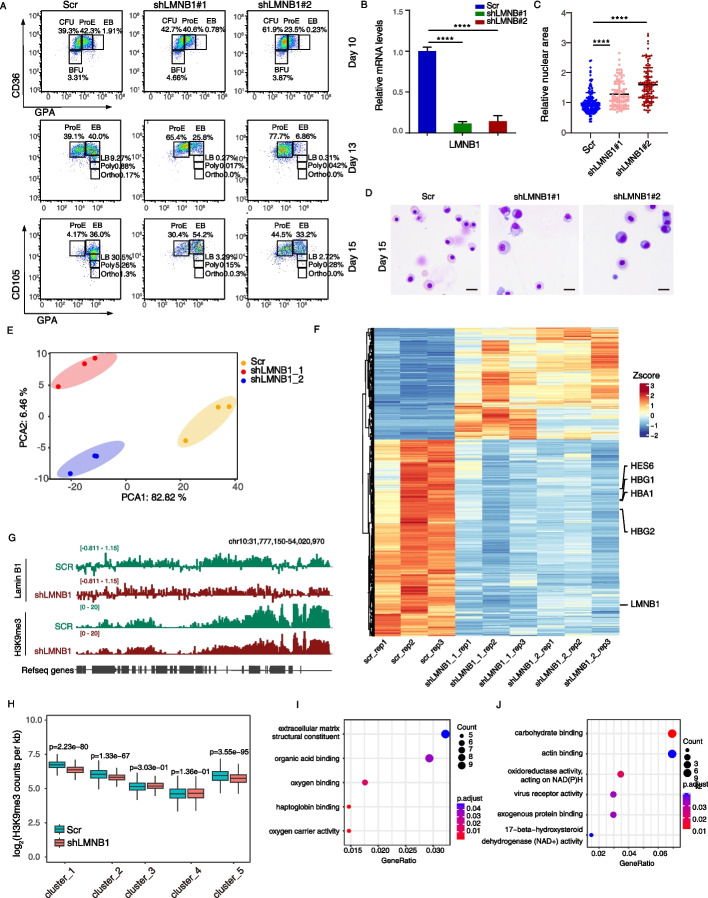
Loss of Lamin B1 impairs heterochromatin establishment and cell differentiation.** A** Flow cytometry analysis shows that *LMNB1* knockdown impairs in vitro erythroid differentiation.Cells were infected with sh*LMNB1* virus on day 7 and collected on days 10, 13, and 15 of erythroid differentiation culture and analyzed by flow cytometry using stage specific surface markers (e.g., CD36, GPA/CD235a, CD105). All major erythroid differentiation stages, BFU-E, CFU-E, ProE, EB, LB, Poly, and Ortho, are labeled in the plots. The relative proportions of each population are indicated. **B** RT-qPCR analysis of LMNB1 mRNA levels in erythroblasts transduced with LMNB1-targeting or scrambled shRNA. Cells were infected at day 7 of differentiation, and GFP-positive cells were sorted at day 12 for RT-qPCR. Data are presented as mean ± SD from at least three independent biological replicates. *P* values were determined by two-tailed Student’s t-test; **** denotes *P* < 0.0001. **C** Quantitative analysis of the MGG staining results showed a greater mean value of nuclei area in LMNB knockdown erythroblasts compared to control. The *p* values were determined by two-tailed Student’s t-test; **** denotes *p* < 0.0001. **D** Wright–Giemsa staining results showing the differences of morphology in erythroblasts differentiation between control and LMNB knockdown cells at day 15. The scale bar represents 20 μm. **E** Results of principal component analysis of *LMNB1* knockdown in erythroblasts compared with controls. Cells were infected at day 7 of differentiation, and GFP-positive cells were sorted at day 12 for RNA-seq analyses. **F** Heatmap showing the relative expression levels of genes that significantly up-regulated/down-regulated in the *LMNB1*knockdown group compared to the control group. **G** Representative genome browser tracks illustrate the changes in Lamin B1 and H3K9me3 signal intensities at selected genomic regions upon *LMNB1* knockdown compared to the scramble control. Cells were infected at day 7 of differentiation, and GFP-positive cells were sorted at day 12 for ChIP-seq analyses. (H) Distribution of H3K9me3 ChIP signal intensities across five chromatin clusters in *LMNB1* knockdown and scramble control cells. The signal intensity is represented as counts per kilobase. The *p* values were determined by two-tailed Student’s t-test. **I** Gene Ontology (GO) enrichment analysis of downregulated genes in *LMNB1* knockdown cells compared to scramble control. **J** Gene Ontology (GO) enrichment analysis of upregulated genes in *LMNB1* knockdown cells compared to scramble control

To investigate the roles of Lamin B1 in differentiation and chromatin compaction, we used lentivirus to knock down *LMNB1* expression on day 7 of differentiation. Lamin B1 knockdown significantly inhibited erythroid differentiation, as confirmed by flow cytometry and Giemsa staining. Additionally, the nuclear volume was significantly larger in the knockdown group compared to the control (Fig. 6A-D). In contrast, knockdown of *LMNA* had only a mild effect on differentiation (Additional file 1: Fig. S7A, B). We conducted RNA-Seq on cells with *LMNB1* knockdown, and observed a significant downregulation of genes involved in erythroid maturation, such as the hemoglobin gene *HBB* and the erythroid-specific transcription factor *HES6* (Fig. 6E, F). Gene Ontology (GO) analysis revealed downregulation of pathways related to oxygen binding, while metabolic pathways, such as oxidoreductase activity, were upregulated (Fig. 6I-J). We also performed Lamin B1 and H3K9me3 ChIP-Seq on erythroblasts, revealing reduced LAD occupancy and decreased levels of H3K9me3 after knockdown (Fig. 6G). Further analysis of different H3K9me3 clusters revealed a reduction in H3K9me3 counts per Kb (Fig. 6H). Taken together, these results suggest that Lamin B1 plays a role in heterochromatin maintenance and is important for normal erythroid differentiation. Together, these findings align closely with previous observations showing that Lamin B degradation occurs specifically at the LB stage, suggesting a potential functional link between nuclear lamina disassembly and the onset of chromatin condensation during terminal erythropoiesis.

## Discussion

**Fig. 7:**
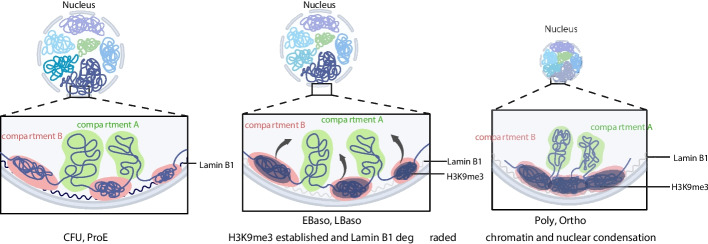
Schematic illustration of Lamin B1-mediated regulation of chromatin organization during erythroid differentiation. During early erythroid differentiation, Lamin B1 contributes to normal differentiation by maintaining LAD stability and nuclear architecture. In the late basophilic stage, loss of Lamin B1 leads to disruption of LADs, which facilitates large-scale chromatin reorganization mediated by heterochromatin. These findings suggest that Lamin B1 serves as a key regulator of higher-order chromatin structure in a stage-specific manner during erythropoiesis

Dynamic changes in heterochromatin play a crucial role in red blood cell development. In this study, we utilized an in vitro erythroid differentiation system to systematically investigate the spatiotemporal evolution of chromatin 3D architecture during terminal erythropoiesis, revealing a chromatin compaction process predominantly associated with by intergenic heterochromatin (Fig. 7).

Heterochromatin marked by histone H3K9 methylation represents a key component of higher-order chromatin organization and plays essential roles in ensuring proper mitotic chromosome segregation, regulating spatial and temporal gene expression, and maintaining genomic stability [32]. By integrating multiple complementary approaches—including Hi-C, ChIP-seq, and immunofluorescence—we provide a more refined and comprehensive map of chromatin conformational dynamics throughout erythroid differentiation.

Our findings are consistent with recent studies showing that H3K9me3-mediated heterochromatin undergoes significant reorganization during erythroid maturation [5]. We observed substantial restructuring of interactions between these heterochromatic regions and the nuclear lamina, suggesting their potential involvement in the spatial reorganization and compaction of chromatin during differentiation.

Importantly, we found that this chromatin compaction process is primarily associated with heterochromatin located in intergenic regions rather than gene-dense regions. Further analysis revealed that Lamin B1, a core component of the nuclear lamina, plays a central regulatory role in this process. Lamin B1 exhibited stage-specific expression and localization patterns: it was broadly distributed along the nuclear envelope at early stages but showed focal loss at later stages, forming distinct heterochromatin-enriched nuclear foci that colocalized with H3K9me3 signals.

Deletion of the long arm of chromosome 5 (5q −) is one of the most common cytogenetic abnormalities in myelodysplastic syndromes (MDS), and the *LMNB1* gene is located at 5q23.2, a core critical region within the commonly deleted segment. Approximately 85% of patients with 5q − MDS exhibit loss of *LMNB1* [33, 34], and refractory anemia is a hallmark clinical feature among these individuals [35], closely mirroring the erythroid maturation defects observed in our experimental models.

## Conclusions

In summary, our study not only uncovers the molecular basis of heterochromatin remodeling during erythroid differentiation, but also highlights the dynamic regulation of Lamin B1 as a potential determinant of red blood cell maturation. Future investigations should focus on whether Lamin B1 deletion directly contributes to erythroid dysfunction and its pathogenic role in MDS, particularly in 5q − -associated subtypes. This line of research may offer novel insights into the interplay between chromatin structural regulation and hematopoietic disorders.

## Methods

### Cell line

The 293 T cell line was obtained from the Cell Bank of the Chinese Academy of Sciences and is routinely monitored for mycoplasma contamination.

### Erythroid differentiation

CD34⁺ hematopoietic stem and progenitor cells were isolated from human umbilical cord blood using magnetic-activated cell sorting (MACS) according to the manufacturer’s instructions. Briefly, mononuclear cells were enriched by density gradient centrifugation, followed by immunomagnetic selection targeting the CD34 antigen. The purified CD34⁺ cells were then used to induce erythroid differentiation using a four-phase liquid culture system. In Phase 1 (Day 0–7), CD34⁺ cells were seeded at a density of 1 × 10⁶ cells/mL in SFEM II medium supplemented with SCF (100 ng/mL), IL-3 (10 ng/mL), IGF-1 (40 ng/mL), IBMX (50 µM), dexamethasone (1 µM), and transferrin (50 µg/mL) to expand the erythroid progenitor population. From Day 7 to Day 13 (Phase 2), cells were transferred to erythroid differentiation medium containing EPO (3 U/mL), reduced SCF (50 ng/mL), transferrin (50 µg/mL), insulin (10 µg/mL), and penicillin/streptomycin (1 ×), while maintaining the cell density at approximately 0.1 × 10⁶ cells/mL. During Phase 3 (Day 13–15), terminal maturation was initiated by supplementing the culture with 5% AB serum, insulin (10 µg/mL), heparin (3 U/mL), transferrin (1 mg/mL), low-dose EPO (1 U/mL), and penicillin/streptomycin (0.5 ×). The cell density was increased to 1 × 10⁶ cells/mL during this stage. In the final phase (Phase 4, Day 15–20), enucleation and terminal differentiation were promoted under similar conditions, except for an increase in cell density to 5 × 10⁶ cells/mL and the removal of EPO. Throughout the differentiation process, cell morphology, viability, and surface marker expression were regularly monitored to assess erythroid commitment and maturation efficiency.

### Isolation of primary human erythroblasts at distinct terminal differentiation stages

During the early stages of differentiation (day 7), erythroid progenitors were classified based on their surface marker expression: CD36⁻GPA⁻ cells were defined as burst-forming unit–erythroid (BFU-E), CD36⁺GPA⁻ as colony-forming unit–erythroid (CFU-E), and CD36⁺GPA⁺ as proerythroblasts (ProE). As differentiation progressed, more mature erythroblast populations emerged. At day 13, GPA^high^ CD105^high^ cells were identified as early basophilic erythroblasts (EB), while GPA^high^ CD105^dim^ cells represented late basophilic erythroblasts (LB). By day 15, terminal differentiation was evident, with GPA^high^ CD105^low^ cells classified as polychromatic erythroblasts (Poly) and GPA^high^ CD105^−^ as orthochromatic erythroblasts (Ortho). Cells at each developmental stage were prospectively isolated by flow cytometry based on these marker combinations and further validated morphologically using Giemsa staining.

### *LMNA *and *LMNB1* knockdown

Two pairs oligos of *LMNA* and *LMNB1* were designed according to specific gene sequence as list in Additional file 3: Table S2, the synthesized oligos were cloned into the pLKO.1 TRC cloning vector using BamHI/KpnI sites. The lentiviruses were generated by co-transfection with pAX2 and pMD2G into 293FT cells. Virus supernatants were collected 2 days later. Then erythroblast cells were transduced, followed by detection with GFP. The target genes expression levels were tested by RT-qPCR.

### Bulk ChIP-seq

ChIP assays were performed on erythroblasts across all developmental stages, including BFU-E, CFU-E, ProE, EB, LB, Poly, and Ortho, following a protocol adapted from previously published methods with minor modifications [36]. Briefly, cells were fixed in 1% formaldehyde for 10 min at room temperature, and the reaction was quenched by adding glycine to a final concentration of 0.125 M for 5 min. Fixed cells were washed twice with ice-cold PBS and pelleted by centrifugation. The cell pellet was resuspended in SDS lysis buffer (1% SDS, 5 mM EDTA, 100 mM NaCl, 0.02% NaN₃, 50 mM Tris–HCl pH 8.1, supplemented with 1 × protease inhibitor cocktail and 1 × PMSF), incubated on ice for 10 min, and nuclei were collected by centrifugation at 1,200 rpm for 6 min. Nuclei were then resuspended in IP buffer (1:1 mixture of SDS buffer and Triton dilution buffer [100 mM Tris–HCl pH 8.6, 100 mM NaCl, 5 mM EDTA, 5% Triton X-100, 0.02% NaN₃, 1 × PIC, and 1 × PMSF]). Chromatin was sonicated to obtain DNA fragments of 200–500 bp in length. After clarification by centrifugation, the lysate was diluted in IP buffer, and an aliquot was reserved as input control. Specific primary antibodies were added, and samples were rotated overnight at 4 °C. On the following day, 30–40 µL of protein A/G magnetic beads were added and incubated for 2–4 h at 4 °C with gentle rotation. Beads were sequentially washed once with low-salt buffer (1% Triton X-100, 0.1% SDS, 150 mM NaCl, 2 mM EDTA, 20 mM Tris–HCl pH 8.0) and three times with high-salt buffer (same components with 500 mM NaCl). Immune complexes were eluted in 120 µL de-crosslinking buffer and incubated at 65 °C for 3 h to overnight with shaking at 1,100 rpm. ChIP and input DNA were purified using the QIAGEN PCR Purification Kit. The DNA Library of ChIP were prepared with a TruePrep DNA Library Prep Kit according to the manufacturer's instructions (TD503, Vazyme). Paired-end 150 bp (PE150) sequencing of the constructed libraries was carried out using the NovaSeq platform (Illumina) by Annoroad.

### BL-Hi-C library generation

All BL-Hi-C libraries were prepared following an adapted protocol based on previously published methods [26], with modifications to enhance efficiency and yield. In brief, the ligation-based BL-Hi-C workflow for HaeIII digestion and one-step proximity ligation included the following key steps: cell fixation, nuclei isolation, in situ restriction enzyme digestion, biotin labeling, and proximity ligation. After ligation, DNA was purified and sheared by sonication prior to library preparation. A major modification involved the direct use of the Vazyme DNA Library Prep Kit (TD607) following biotin pull-down, which significantly improved both the yield and quality of the final libraries compared to conventional approaches. The resulting libraries were amplified via PCR and sequenced using the NovaSeq platform (Illumina) by Annoroad. This optimized protocol facilitated robust detection of chromatin interactions across all erythroid developmental stages analyzed.

### Immunofluorescence

As described previously [37], for immunofluorescence analysis, erythroblasts at defined developmental stages were cytospun onto glass slides and fixed with cold methanol for 3 min. Following fixation, samples were washed twice with PBS and blocked with 0.8% BSA for 10 min at room temperature to reduce nonspecific binding. Primary antibodies were diluted in PBS and applied to the samples, followed by incubation at 37 °C for 1 h in a humidified chamber. After three washes with PBS, fluorescent secondary antibodies were added and incubated under the same conditions for an additional hour. Nuclei were counterstained with DAPI. Fluorescent images were acquired using a laser scanning confocal microscope. Image processing and signal quantification were performed using CellProfiler image analysis software. We first identified nuclei using IdentifyPrimaryObjects. H3K9me3 puncta were then enhanced with EnhanceOrSuppressFeatures (Speckles mode), and analysis was restricted to the nuclear region using a nucleus derived mask. H3K9me3 speckles within this mask were segmented using IdentifyPrimaryObjects, and their size and shape features (e.g., area) were quantified by MeasureObjectSizeShape. Detailed information on antibodies, is provided in the Additional file 3: Table S2.

### Chromosome painting

For chromosome painting analysis, cells were cultured on 22 × 22 mm^2^ coverslips until reaching 70–80% confluency. Cells were then fixed in a freshly prepared cold fixative solution of methanol and acetic acid (3:1 ratio) at − 20 °C for 20 min. Following fixation, samples were air-dried and hybridized with a probe mix containing 5 µL each of directly labeled fluorescent DNA probes specific for human chromosomes 2 and 19 (MetaSystems), resulting in a total hybridization volume of 10 µL. The coverslip was carefully placed face-down onto the probe mixture and sealed with rubber cement to prevent evaporation. Co-denaturation of chromosomal DNA and probes was achieved by heating the slide on a hot plate at 75 °C for 2 min. Hybridization was carried out overnight at 37 °C in a humidified chamber to ensure optimal binding. After hybridization, the coverslip was gently removed and washed sequentially in pre-warmed 0.4 × SSC at 72 °C for 2 min, followed by room temperature washing in 2 × SSC with 0.05% Tween-20 for 30 s. Slides were briefly rinsed in PBS to remove residual detergent and mounted using ProLong® Diamond Antifade Mountant with DAPI (P36962, Thermo Fisher Scientific) for nuclear counterstaining and fluorescence preservation. Fluorescent signals were visualized using a laser scanning confocal microscop.

### Giemsa staining

Cells were harvested at the indicated stages and cytospun onto glass slides using a cytocentrifuge. The slides were air-dried completely at room temperature and then fixed in methanol for 3 min at room temperature. The slides were stained in Giemsa staining kit (Solarbio G4640), Excess stain was removed by rinsing briefly with distilled water. Cell morphology was examined under a light microscope, and images were captured for morphological analysis.

### ChIP-seq data processing

Raw sequencing reads were quality-filtered and adapter-trimmed using fastp. Clean reads were aligned to the human reference genome (GRCh38/hg38) using Bowtie2, and duplicate reads were removed with SAMtools. Only uniquely mapped reads with a mapping quality score ≥ 30 were retained for downstream analysis. Peak calling was performed using MACS2 with default settings. For visualization and quantitative analysis, normalized bigwig files were generated using deepTools with RPKM normalization and a bin size of 10 bp. The Fraction of Reads in Peaks (FRiP) was calculated to evaluate overall ChIP-seq signal quality. Peak annotation and association with genomic features were performed using ChIPseeker.

### Chromatin state characterization

To characterize the chromatin states across erythroid differentiation stages, we integrated multiple histone modification profiles, including H3K9me3, H3K27me3, H3K27ac, H2AK119ub, and H3K4me1, using the ChromHMM pipeline. Briefly, aligned ChIP-seq data for each mark were binarized across the genome based on signal enrichment, and hidden Markov models were applied to learn combinatorial chromatin patterns. This approach enabled us to define a set of recurrent chromatin states that reflect distinct functional elements, such as active promoters (marked by H3K4me1 and H3K27ac), enhancers (H3K27ac-high/H3K4me1-high), bivalent domains (H3K4me1/H3K27me3 co-enriched), constitutive heterochromatin (H3K9me3-dominant), and Polycomb-repressed regions (H3K27me3 and H2AK119ub enriched). The resulting state annotations were used to explore dynamic changes in chromatin organization during erythropoiesis and their association with gene expression and nuclear compartmentalization.

### LAD calling

Lamina-associated domains (LADs) were identified using the Enhanced Domain Detection (EDD) algorithm, which defines nuclear lamina-interacting regions based on enrichment of ChIP-seq signal relative to input. LADs were called at a resolution of 20 kb with Gaussian smoothing across 5 bins (-g 5). The log2 ratio of ChIP over input signal was calculated and outputted for downstream visualization (–write-log-ratios). Chromosome sizes and alignment parameters were specified using the hg38 reference genome file, together with a custom configuration file containing filtering thresholds such as FDR cutoff and minimum domain length. Input-normalized Lamin B1 ChIP-seq data from individual samples were used for LAD identification. LADs were directly used for downstream annotation and comparison with chromatin state segmentation derived from ChromHMM, aiming to explore their potential epigenetic roles during erythroid differentiation.

### RNA-seq data processing

RNA-seq data were processed using fastp for adapter trimming and quality filtering. Paired-end reads were subjected to stringent quality control, with a minimum Phred score of 30 and a read length cutoff of 50 bp. Adapter sequences were removed, and only high-quality paired reads were retained for downstream analysis. Cleaned reads were aligned to the human reference genome (GRCh38/hg38) using STAR, allowing for accurate transcriptome mapping and splice junction detection. Transcript-level and gene-level expression quantification was performed using RSEM, generating both TPM-normalized values and raw read counts. Expression estimates across samples were aggregated into matrices using rsem-generate-data-matrix for subsequent differential expression and clustering analyses.

### BL-Hi-C data processing

BL-Hi-C libraries were processed using a combination of trimLinker [26] and HiC-Pro [38] for high-resolution chromatin interaction analysis. Briefly, paired-end raw reads were first subjected to adapter trimming using trimLinker with the following parameters: -m 1 -k 2 -e 1, and custom linker sequences (A: ACGCGATATCTTATC, B: AGTCAGATAAGATAT). Cleaned reads were then used for downstream alignment and processing. Read pairs were aligned and iteratively corrected using HiC-Pro (v2.7.1b) as previously described (ref). Sequencing reads were independently aligned to the human reference genome (GRCh38/hg38) using Bowtie2 in end-to-end mode with the –very-sensitive option. To recover chimeric fragments spanning the ligation junction, the ligation site was detected, and the 5′ portion of each read was realigned to the reference genome. Reads that failed to align, mapped to multiple locations, or were classified as singletons were discarded. Aligned read pairs were assigned to HaeIII restriction fragments. Artifactual interactions, including uncut DNA, self-circularization events, and PCR duplicates were filtered out. Valid inter-ligation read pairs involving two distinct restriction fragments were retained for downstream analysis.Valid read pairs were binned at specific resolutions by dividing the genome into equal-sized bins. We used 100-kb and 200-kb bin sizes to examine global chromatin interaction patterns and 50-kb bin size for local interaction analyses and TAD calling. Interaction matrices were normalized using the Knight-Ruiz (KR) correction method to account for biases such as GC content, mappability, and restriction fragment length. The resulting contact matrices were further converted into.hic format for visualization using tools such as Juicebox. The ‘triangle’ interaction heatmaps to show TADs were generated with 3D Genome Browser (https://epigenomegateway.wustl.edu/).

### Validation of BL-Hi-C data

To assess the consistency between our Hi-C data and previously published datasets, as well as across biological replicates, we calculated pairwise Pearson correlation coefficients based on chromatin interaction frequencies. Interaction counts were binned at 50-kb and 250-kb resolutions and normalized for sequencing depth to minimize technical bias. Pearson correlations were then computed by comparing signal intensities across all sample pairs, providing a quantitative measure of reproducibility and global similarity in chromatin interaction patterns.

### Analysis of TADs

Topologically Associating Domains (TADs) were identified using the hicFindTADs algorithm from the HiCExplorer toolkit [39], which quantifies chromatin insulation through a TAD-separation score. This score is defined as the mean z-score of inter-bin contact frequencies between upstream and downstream genomic regions within a sliding window of variable size. Local minima in this score correspond to putative TAD boundaries. The method was applied to KR-normalized cis-contact matrices derived from BL-Hi-C data at 50-kb resolution. TAD calling was performed with a minimum domain size of 150 kb and a maximum of 500 kb, using a step size of 50 kb to allow fine-grained boundary detection. A delta parameter of 0.01 was used for boundary delineation, and statistical significance was evaluated without correction for multiple testing to maintain sensitivity. These parameters enabled robust identification of TAD structures across all samples.

### Identification of chromatin compartments

To define A/B compartments from Hi-C data at 50-kb resolution, we computed the first principal component (PC1), also known as the eigenvector, using Juicer Tools [40]. PC1 reflects the global organization of chromatin into two major compartments based on intra- and inter-compartmental interaction patterns. For each chromosome, the sign of the eigenvector was manually curated to ensure biological consistency across samples: regions with positive values were classified as compartment A (typically associated with active, euchromatic domains), whereas negative values were assigned to compartment B (corresponding to inactive, heterochromatic regions). This manual adjustment was guided by known genomic features, gene expression patterns, and histone modification profiles to preserve functional interpretability. The resulting eigenvector profiles were used for downstream analyses, including correlation with epigenetic marks, LAD annotations, and nuclear lamina interactions during erythropoiesis.

### Analysis of compartment strength

The resulting eigenvector tracks were then used to compute compartment strength by calculating the mean intra-bin interaction enrichment as a function of genomic distance. This was achieved using the compartment_strength function [41], which quantifies the degree of segregation between regions of similar compartment identity. The resulting saddle plots reflect the extent of preferential interactions within and between high- and low-signal regions, providing a global view of chromatin phase separation and functional compartment organization.

### PlotDistVsCounts

Briefly, for each chromosome, pairwise interactions were binned based on their genomic distance into logarithmic intervals, and the total number of interactions within each bin was calculated. The resulting plot reflects the expected decay of interaction frequency with increasing genomic distance, which serves as a quality control metric and provides insights into the overall chromatin organization. This approach was applied to KR-normalized contact matrices at 50-kb resolution to ensure consistency with downstream analyses such as compartment calling and TAD detection. The generated distance–interaction plots were used to compare global chromatin behavior across different erythroid developmental stages and experimental conditions.

### 3D genome modeling

Topologically associating domains (TADs) were identified using the Arrowhead algorithm implemented in Juicer Tools, based on KR-normalized intra-chromosomal contact matrices at 50-kb resolution. A minimum domain size of 2 Mb was applied to ensure robust detection of structurally coherent TADs across all autosomes and the X chromosome. Domain boundaries were annotated using hg38 reference genome coordinates for downstream analysis. Intra- and inter-chromosomal interactions were analyzed at 50-kb and 1-Mb resolution, respectively, with centromeric regions excluded due to mapping ambiguity. Interaction intensities were evaluated using the Normalized Contact Heatmap Generator (NCHG), and statistical significance was assessed after Benjamini–Hochberg correction for multiple testing (FDR < 0.01). Three-dimensional chromatin structures were reconstructed using Chrom3D [42] by integrating normalized interaction data with physical constraints. The modeling parameters included a compression factor of 0.15, radius of 5.0, and 2,000,000 iterations, generating diploid models that were exported in.cmm format for visualization in UCSF ChimeraX. All interaction data were compiled into a comprehensive.gtrack file for integration and downstream analysis.

### Peripheral, center, and inter assignment in the modeled structures

To define nuclear subcompartments—center, intermediate, and peripheral—based on spatial positioning in 3D chromatin models, the geometric center of each nucleus was first determined as the centroid of all genomic bead coordinates from.cmm files generated by Chrom3D. For each bead, its Euclidean distance to this center was calculated using its 3D Cartesian coordinates (x,y,z), based on the formula:

$$Distance = \sqrt{{x}^{2}+ {y}^{2}+ {z}^{2}}$$

The nuclear radius for each model was then defined as the average distance of the farthest 5% of beads from the center, representing the approximate boundary of the nucleus. Final nuclear radius values were obtained by averaging across all models after quality filtering, with only models containing at least 20 valid bead positions retained to ensure structural reliability. Outliers were removed using standard deviation-based filtering, and the mean nuclear radius, along with its range and standard deviation, was recorded.

Genomic beads were classified into one of three spatial compartments based on their relative distance to the nuclear center: Peripheral: Beads located at > 80% of the nuclear radius, corresponding to regions near the nuclear lamina; Center: Beads located at < 20% of the radius, representing the most internal chromatin regions; Intermediate: Beads located between 20 and 80% of the radius, encompassing the transitional space. Heatmaps were generated using the Spectral_r color map, where red indicates nuclear center localization and blue corresponds to nuclear periphery; LAD annotations were overlaid for heterochromatin validation. We generated 400 distinct 3D chromatin models and used an averaged representation to capture the general spatial organization while minimizing structural noise from individual realizations. Data integrity was ensured by excluding invalid coordinates (e.g., NaN values) and outlier distances.

### Datasets used in this study

ChIP-Seq, RNA-seq and BL-Hi-C data that support the findings of this study have been deposited in the CNCB (https://ngdc.cncb.ac.cn/) under accession codes HRA012152 [43].

RNA-seq data in erythropoiesis development were from previous publication (GSE107218 [44]) with quality control filtration. Code for data processing and analysis is available on GitHub [45] and archived on Zenodo [46] under the MIT License (https://opensource.org/licenses/MIT). Original images have been deposited to Figshare and are publicly accessible at https://doi.org/10.6084/m9.figshare.31238848 [47].

## Supplementary Information

Additional file 1: Supplementary figures (Additional file 1: Figs. S1-S9).

Additional file 2: Table S1. Quality control metrics for BL-HiC library sequencing alignment.

Additional file 3: Table S2. Table of reagent/resource catalog number and software version.

## Data Availability

ChIP-Seq, RNA-seq and BL-Hi-C data that support the findings of this study have been deposited in the CNCB under accession codes [HRA012152] (https://ngdc.cncb.ac.cn/gsa-human/browse/HRA012152). The imaging data have been deposited in Figshare (DOI: 10.6084/m9.figshare.31238848). All constructs and cell lines generated or used in this study are available from the lead contact upon reasonable request. Code for data processing and analysis is available on GitHub and archived on Zenodo under the MIT License (https://opensource.org/licenses/MIT). Requests for further information, materials, or resources should be directed to the lead contact, Dawei Huo (huodawei@zju.edu.cn).
