# Supplementary material for: Dynamic changes in Lamin B1 and heterochromatin coincide with chromatin condensation during human erythropoiesis
Source: Genome Biol. 2026 Mar 24;27:148. doi: 10.1186/s13059-026-04025-x (PMC13134142; doi:10.1186/s13059-026-04025-x)
Supplement: Supplementary file 1 — Additional file 1: Supplementary figures (Additional file 1: Figs. S1-S9). [file 13059_2026_4025_MOESM1_ESM.pdf]

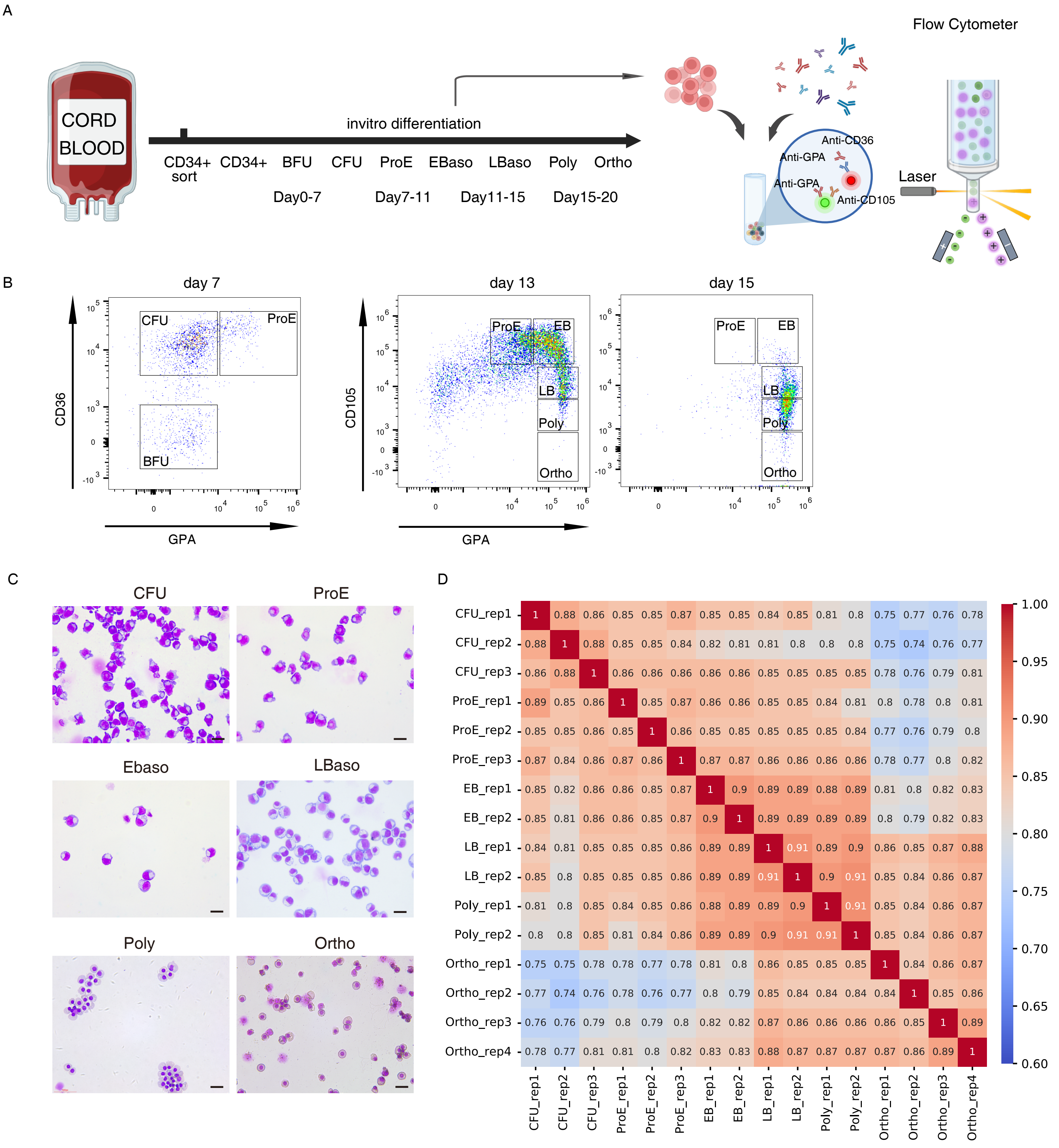

Figure S1. Workflow and validation of in vitro erythroid differentiation and chromatin conformation analysis.
(A) Overview of the process of in vitro isolation of umbilical cord blood, differentiation and flow sorting to obtain cells.
The relative relationship between the number of days of erythriod differentiation and the stage of differentiation is also shown
(B) Results of flow cytometry analysis of different differentiation stages. (Defined using anti-CD36 and anti-GPA for early stage of differentiation and anti-CD105 and anti-GPA for middle to late stage of differentiation)
(C) Wright–Giemsa staining showing the morphology of erythroblasts at different differentiation stages under light microscopy, the scale bar represents 20 μm.
(D) A Pearson correlation heatmap was generated based on genome-wide chromatin interaction matrices from all BL-HiC samples.

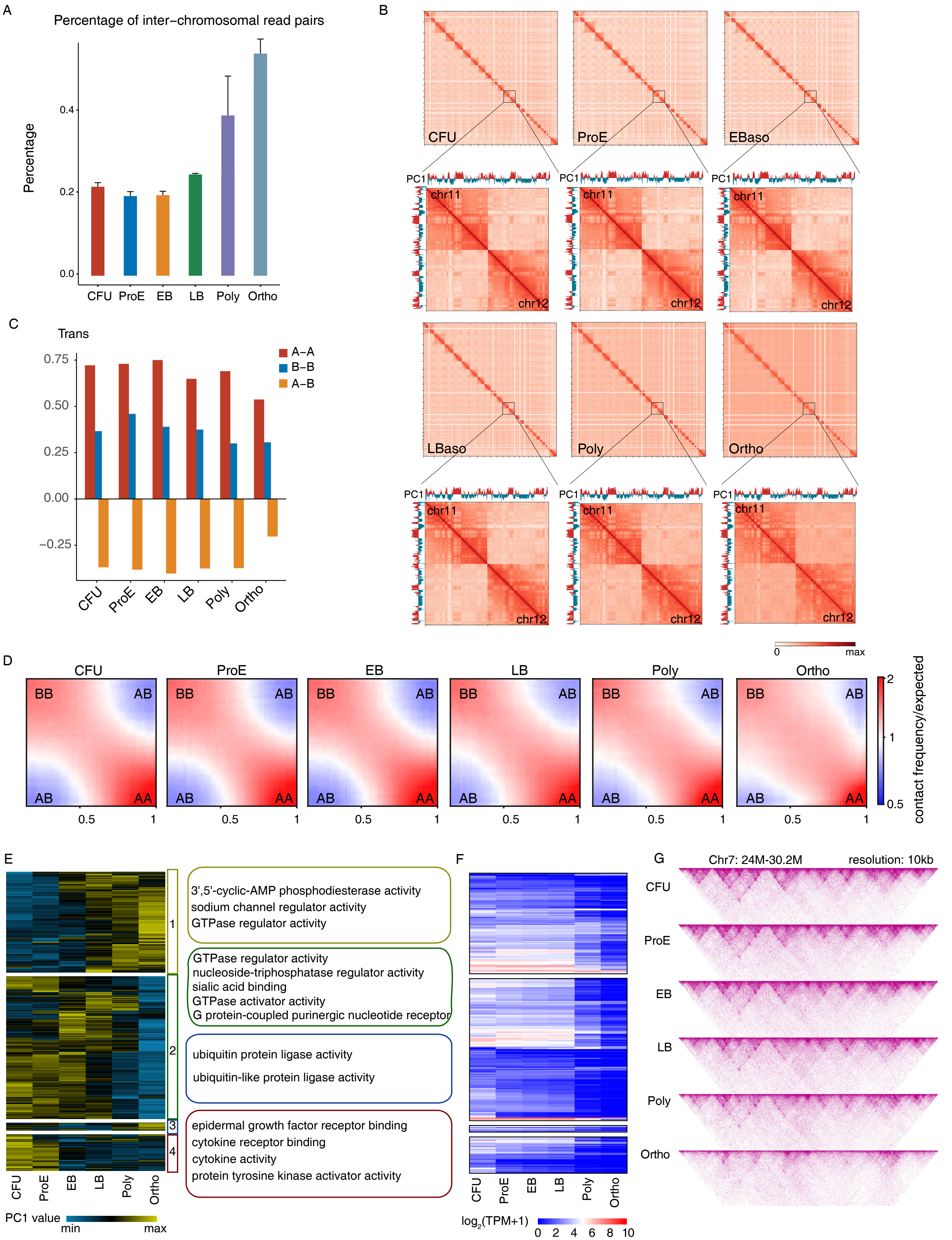
A
Percentage of inter−chromosomal read pairs
Percentage
0.0
0.2
0.4
CFU ProE EB LB Poly Ortho
B
CFU
ProE
EBaso
LBaso
Poly
Ortho
PC1
chr11
chr12
0
max
C
Trans
A−A
B−B
A−B
0.75
0.50
0.25
0.00
−0.25
CFU ProE EB LB Poly Ortho
D
CFU ProE EB LB Poly Ortho
BB AB
AB AA
0.5 1
2
1
0.5
contact frequency/expected
E
1
2
3
4
3',5'-cyclic-AMP phosphodiesterase activity
sodium channel regulator activity
GTPase regulator activity
GTPase regulator activity
nucleoside-triphosphatase regulator activity
sialic acid binding
GTPase activator activity
G protein-coupled purinergic nucleotide receptor
ubiquitin protein ligase activity
ubiquitin-like protein ligase activity
epidermal growth factor receptor binding
cytokine receptor binding
cytokine activity
protein tyrosine kinase activator activity
CFU ProE EB LB Poly Ortho
PC1 value
min
max
F
CFU ProE EB LB Poly Ortho
log2(TPM+1)
0 2 4 6 8 10
G
Chr7: 24M-30.2M
resolution: 10kb
CFU
ProE
EB
LB
Poly
Ortho

Figure S2. Dynamic changes in inter-chromosomal interactions and compartment organization during erythroid differentiation.
(A) Percentage of inter-chromosomal reads pairs at different stages of differentiation.
(B) Contact matrix of the entire genome of erythroblasts at different stages of differentiation, with local zoom in to show chromosomes 11 and 12, and the corresponding PC1 values of the regions.
(C) Continuity of different compartment trans-interaction at different stages of differentiation in the erythroid lineage.
(D) Heatmap showing different compartment A/B trans-interactions at different stages of differentiation in the erythroid lineage.
(E) Heatmap showing genomic regions exhibiting A/B compartment switches during differentiation, clustered by their dynamics. Genes associated with these regions were subjected to GO functional enrichment analysis, and the significantly enriched pathways are annotated on the right. The color scale represents the scaled PC1 values.
(F) Heatmap showing the expression dynamics of genes located within A/B compartment-switching regions. The color scale represents $\log_2$(TPM + 1) values.
(G) Heatmaps showing normalized Hi-C interaction frequencies (10-kb bin) of chromosome 7: 24M-30.2M at different stages of erythroid differentiation.

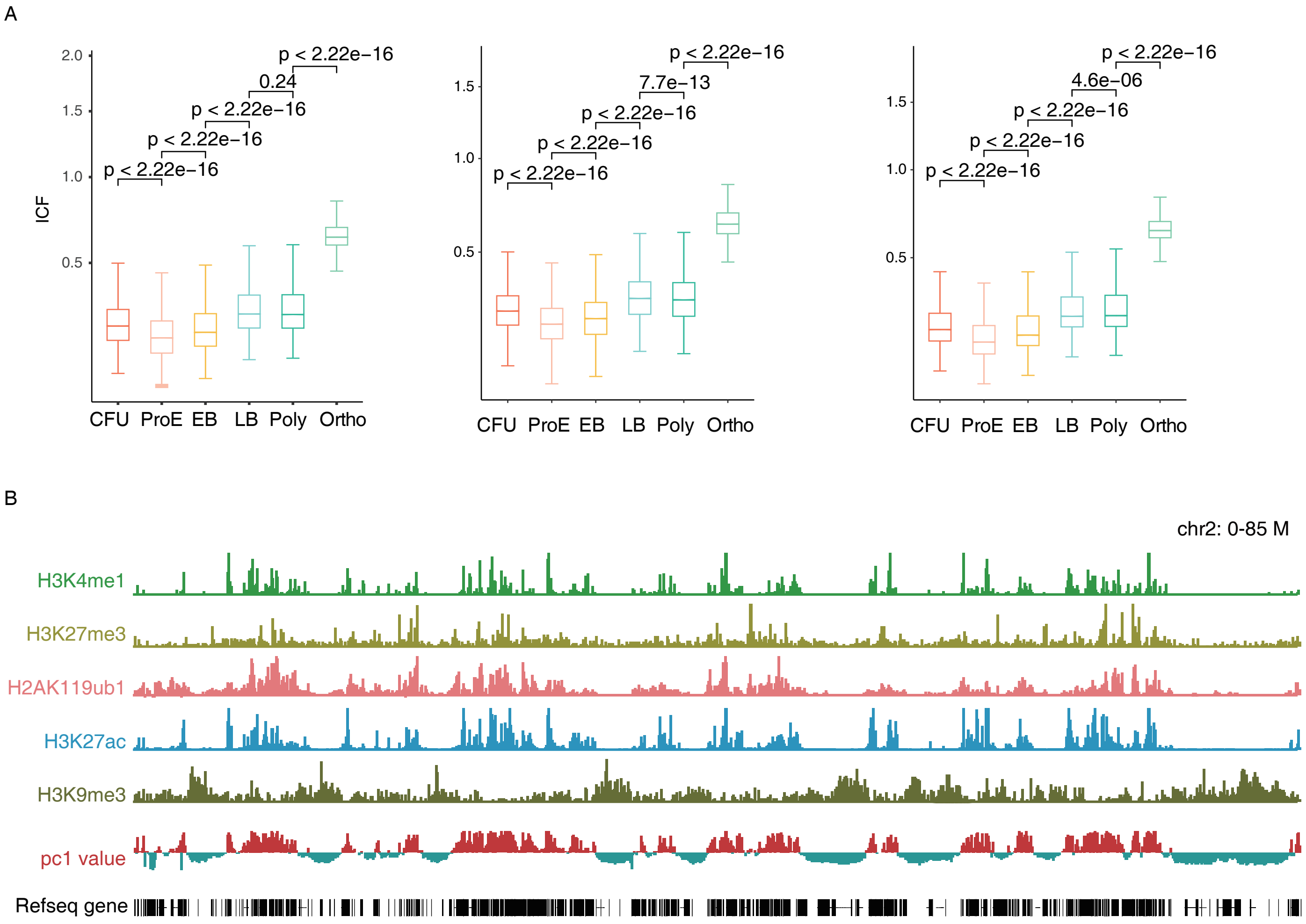

Figure S3. Stage-specific ICF and compartment-associated histone marks.
(A) Changes in ICF (Interchromosomal Fraction of Interactions), with erythroid differentiation in different regions of the chromosome (whole genome(left), compartment A(middle), compartment B(right)). The p-value is also shown.
(B) Representative tracks illustrate H3K4me1, H3K27me3, H2AK119ub, H3K27ac, H3K9me3 ChIP signals in A/B compartments, defined by PC1 values from Hi-C data in Ortho stage.

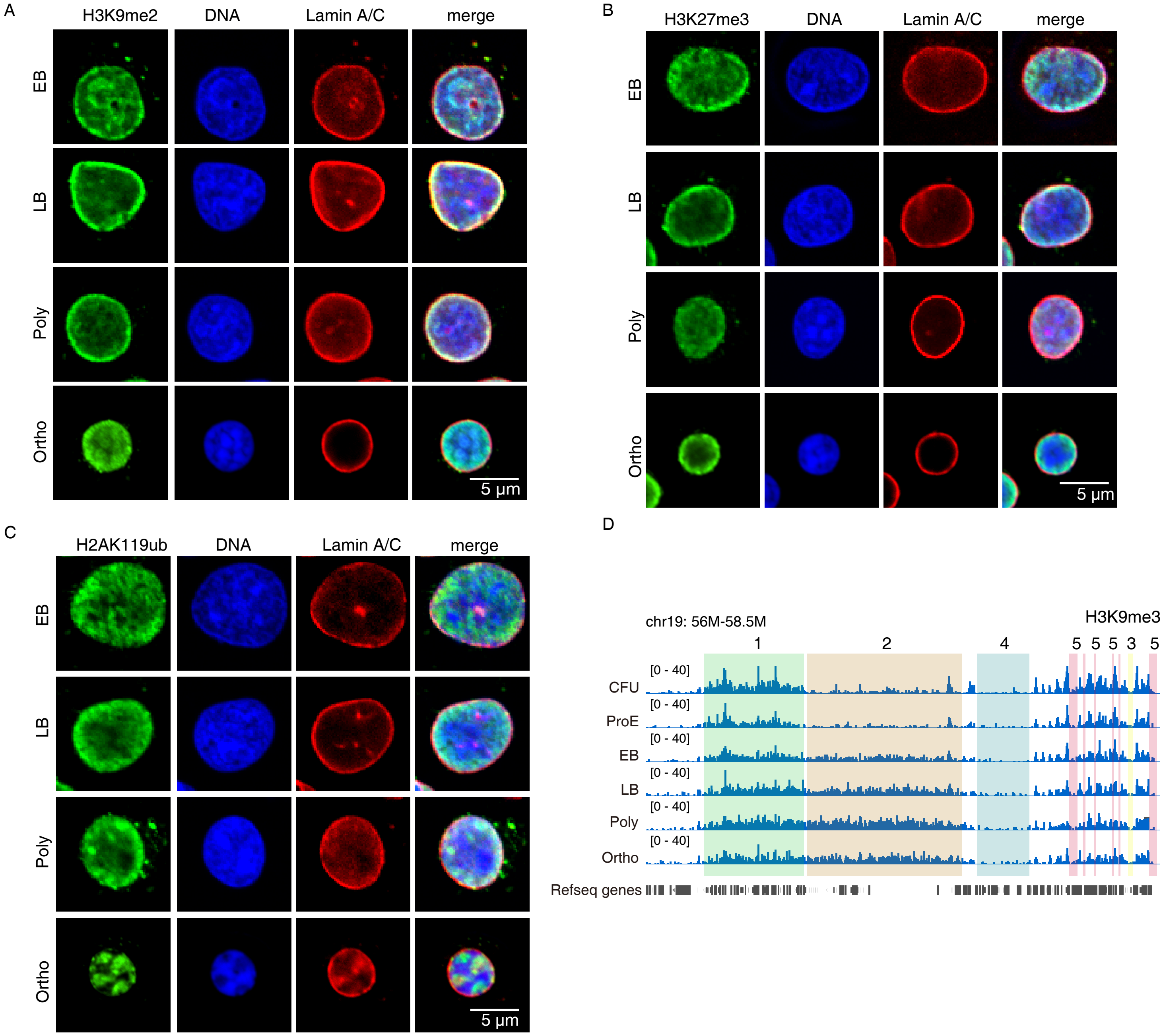

Figure S4. Dynamic reorganization of repressive histone marks during erythroid differentiation.
(A) Immunofluorescence showing changes of H3K9me2 in the nucleus at different stages of erythroid differentiation.
(B) Immunofluorescence showing changes of H3K27me3 in the nucleus at different stages of erythroid differentiation.
(C) Immunofluorescence showing changes of H2AK119ub in the nucleus at different stages of erythroid differentiation.
(D) Representative tracks illustrate stage-specific alterations in H3K9me3 signal within each cluster during erythropoiesis, revealing shifts in heterochromatin organization associated with differentiation progression.

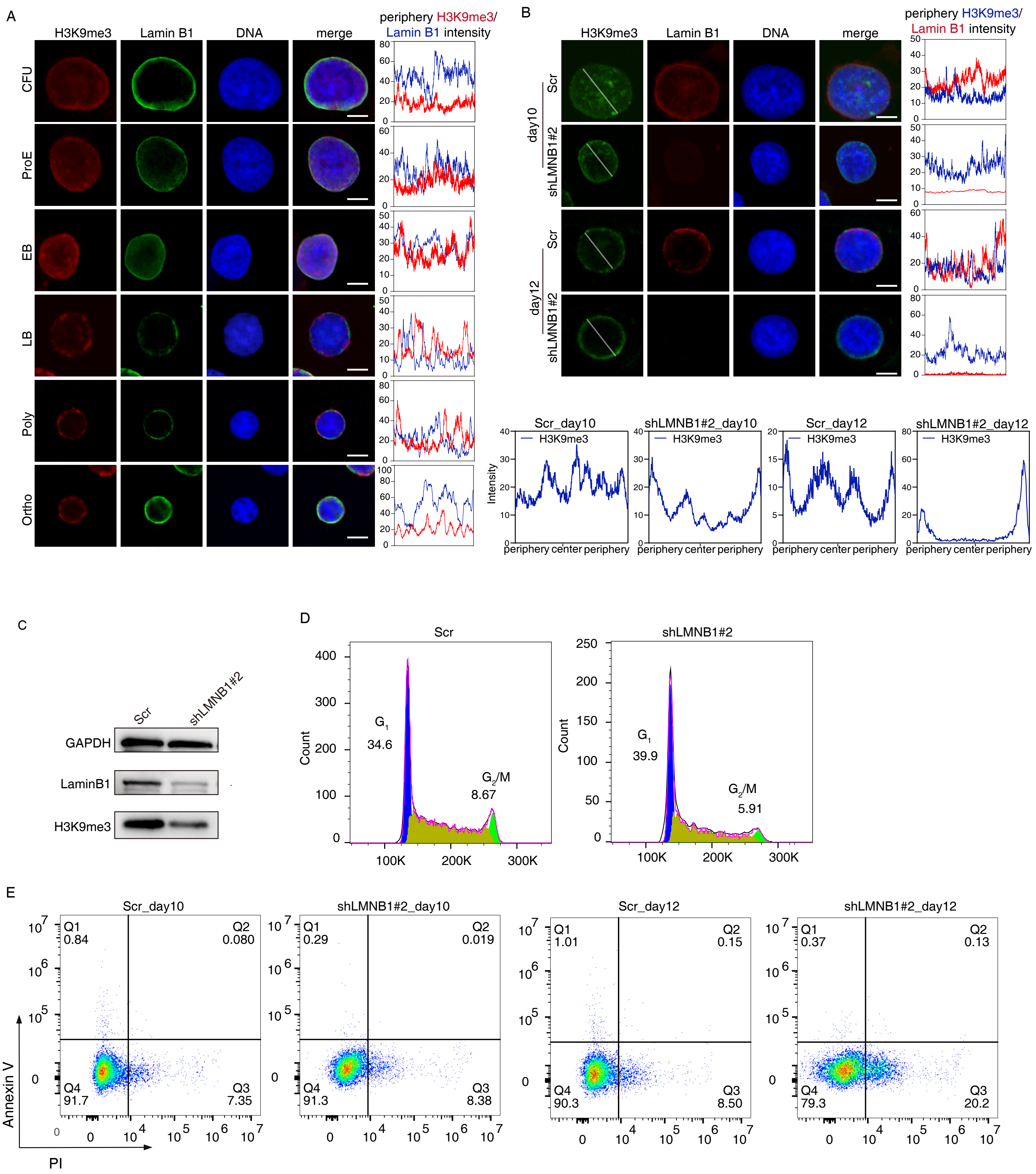

Figure S5. Functional analysis of Lamin B1 and H3K9me3 in erythroid differentiation.

(A) Immunofluorescence analysis of H3K9me3 and Lamin B1 distribution during erythroid differentiation. Quantitative analysis of fluorescence intensities along the entire nuclear periphery is presented on the right, where the x-axis represents circumferential position (0°–360°) and the y-axis represents normalized fluorescence intensity.

(B）Immunofluorescence analysis of H3K9me3 and Lamin B1 distribution during erythroid differentiation in control and LMNB1 knockdown group at day10 and day12 within infected at day7. Quantitative analysis of fluorescence intensities along the entire nuclear periphery is presented on the right, where the x-axis represents circumferential position (0°–360°) and the y-axis represents normalized fluorescence intensity, radial intensity profiles of H3K9me3 measured along a line traversing from nuclear periphery to center is presented on the bottom.

(C) Western blot analysis of H3K9me3 and Lamin B1 in control and LMNB1 knockdown group at day12 within infected at day7.

(D) Cell cycle analysis by flow cytometry in control and LMNB1 knockdown cells (infected on day7) at day12.

(E) Flow cytometry analysis of Annexin V and propidium iodide (PI) staining in control and LMNB1 knockdown cells (infected on day7) at day10 and day12 to assess apoptosis and cell viability.

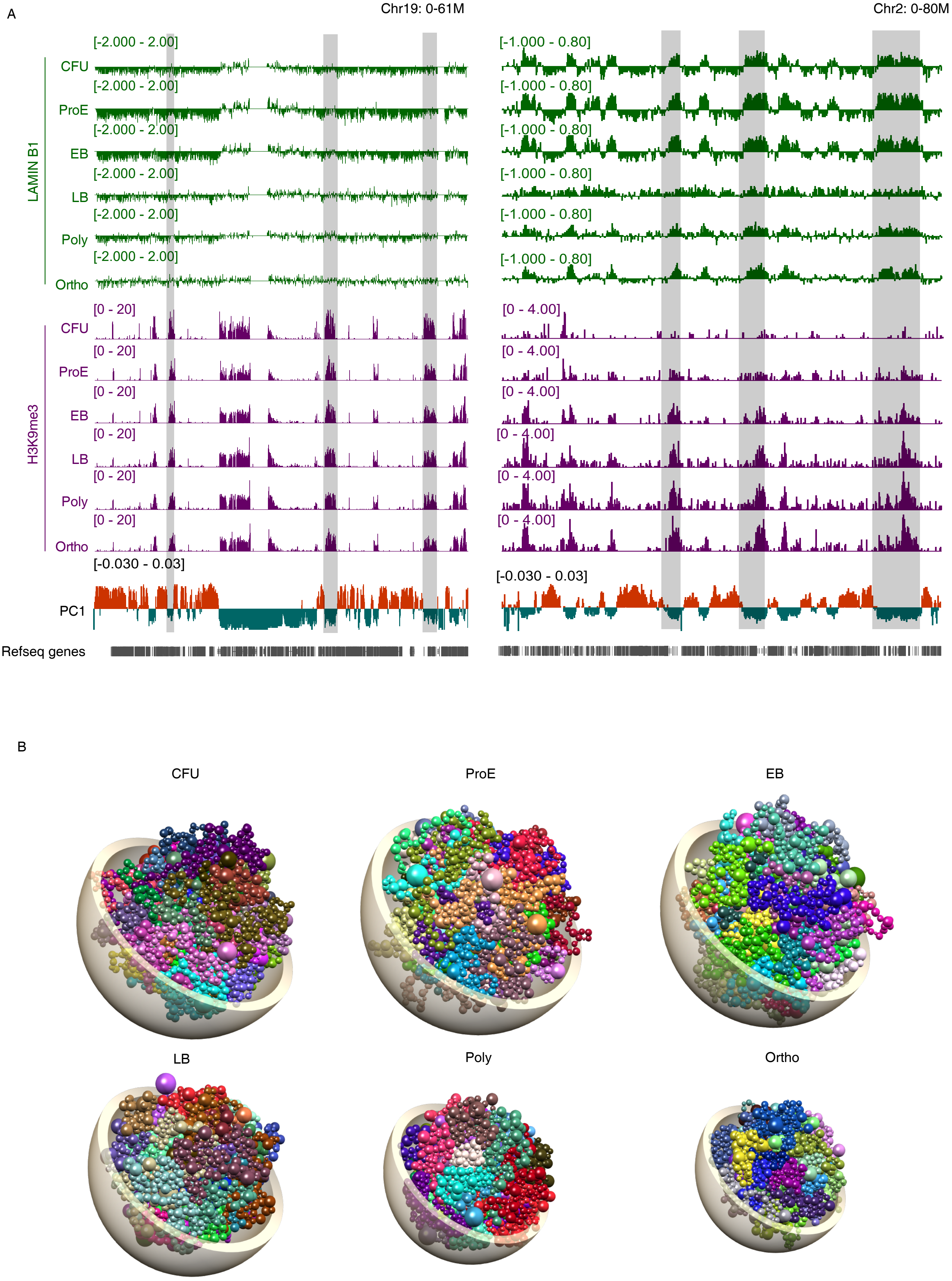

A
Chr19: 0-61M
Chr2: 0-80M
LAMIN B1
CFU
ProE
EB
LB
Poly
Ortho
[-2.000 - 2.00]
[-1.000 - 0.80]
H3K9me3
[0 - 20]
[0 - 4.00]
PC1
[-0.030 - 0.03]
Refseq genes
B
CFU
ProE
EB
LB
Poly
Ortho

Figure S6. Lamin B1 and H3K9me3 distribution in A/B compartments and 3D chromatin reorganization during erythropoiesis.
(A) Genome browser tracks show the distribution of Lamin B1 and H3K9me3 signals in A/B compartments, defined by PC1 values from Hi-C data.
(B) Chromosome-resolved 3D genome models were generated using Chrom3D, with each chromosome represented in a distinct color. Structures from representative stages are shown, revealing stage specific chromatin reorganization and spatial repositioning of chromosomes during erythropoiesis.

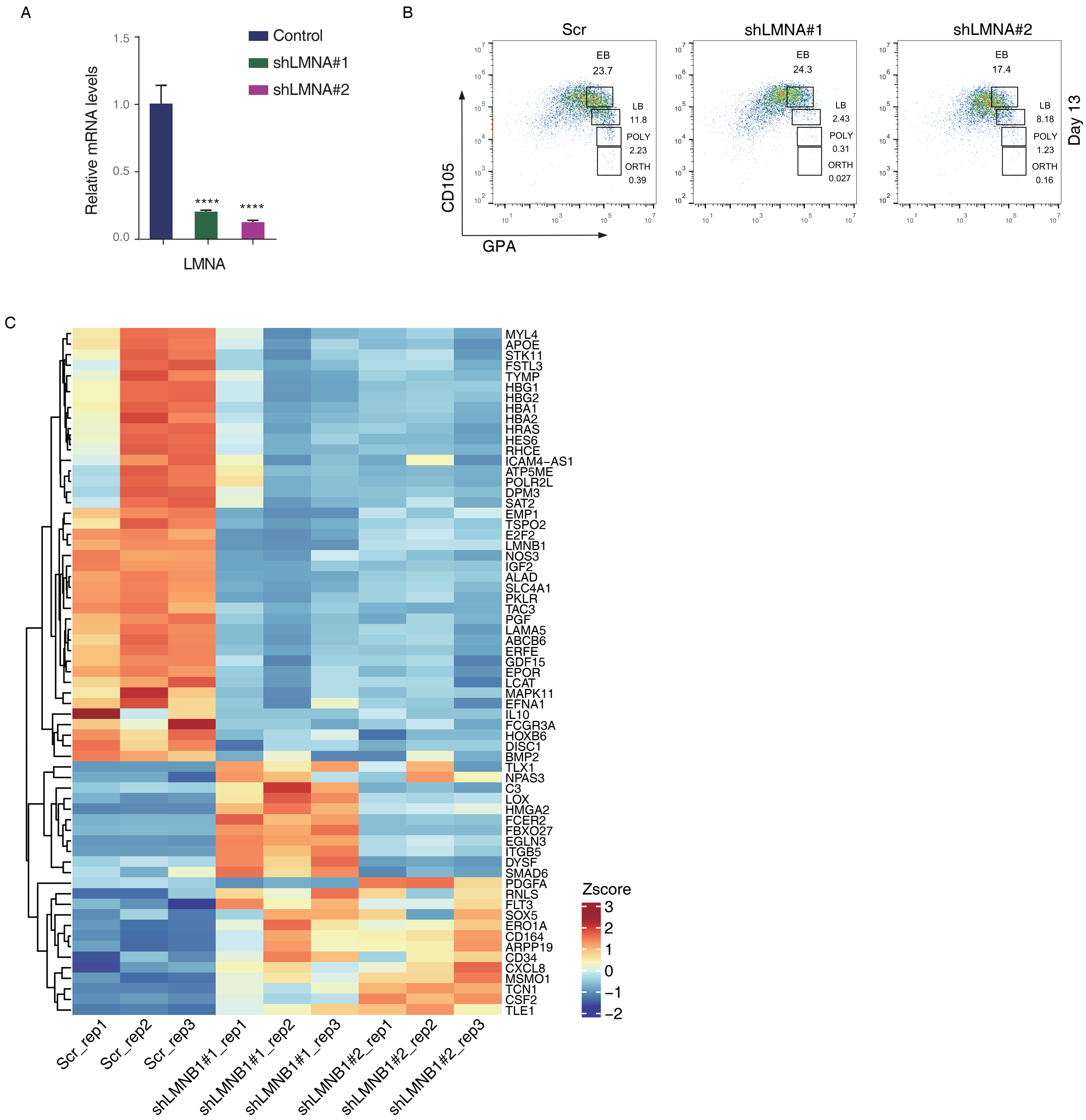

Figure S7. LMNB1 knockdown affects the expression of erythropoiesis related genes.
(A) qPCR analysis of LMNA knockdown efficiency in erythroblasts. Cells were infected at day 7 of differentiation, and GFP-positive cells were sorted at day 12 for RT-qPCR. Data are presented as mean ± SD from at least three independent biological replicates. P values were determined by two-tailed Student's t-test; **** denotes $P < 0.0001$. (B) Flow cytometry showing that LMNA knockdown slightly suppressed in vitro erythroid differentiation of cord blood-derived HSCs at the early stage.
(C) The heatmap displays significantly differentially expressed genes associated with erythropoiesis (curated from GeneCards) upon LMNB1 knockdown. The color intensity represents z-scores.

H2AK119ub

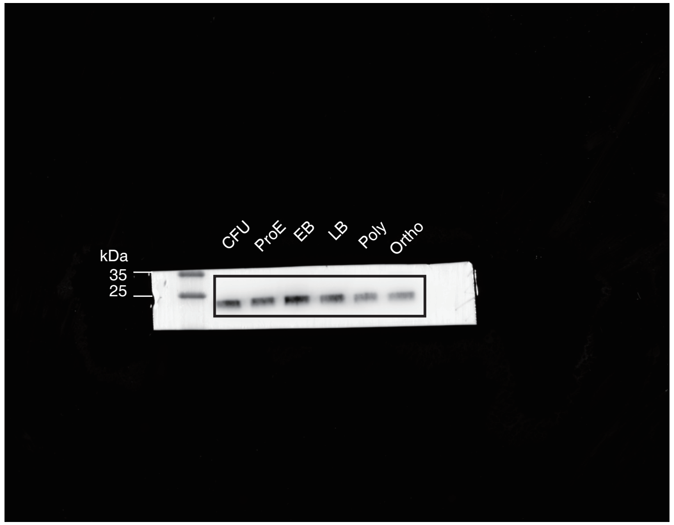

H3K4me1

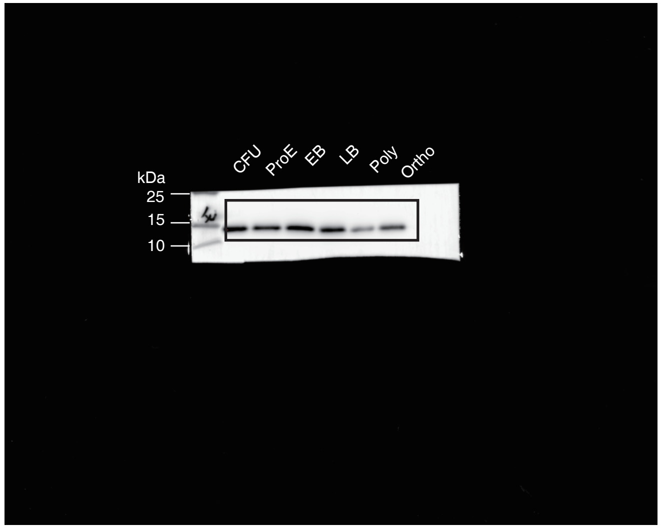

H3K9me2

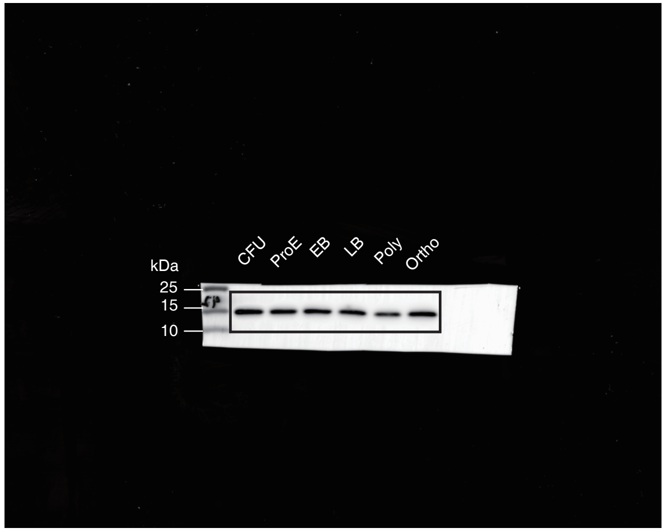

H3K9me3

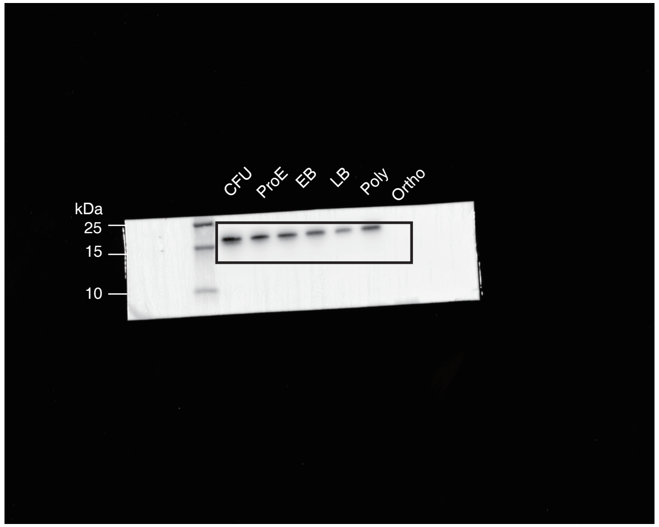

H3K27ac

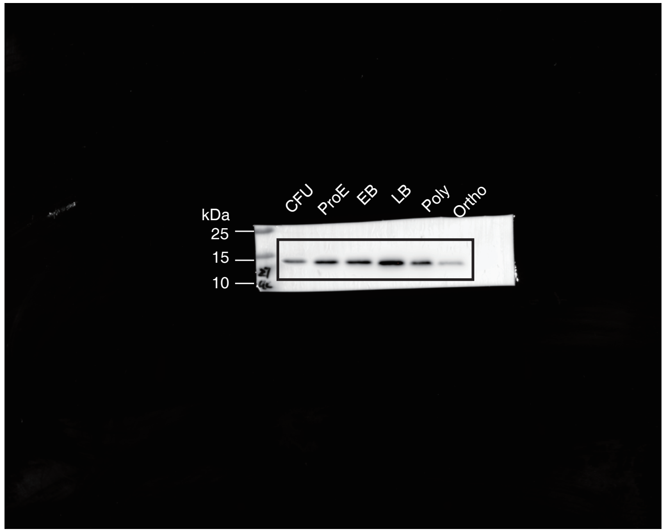

H3K27me3

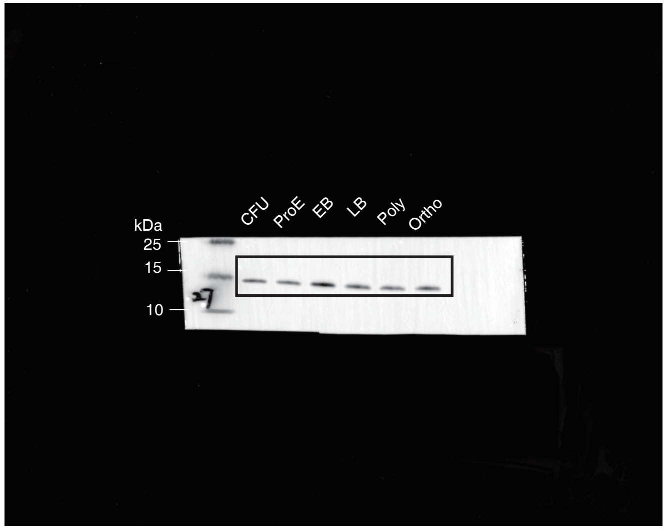

H4K20me1

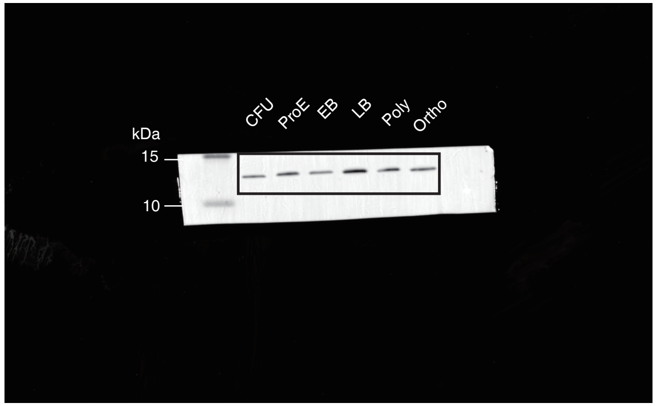

H4K20me3

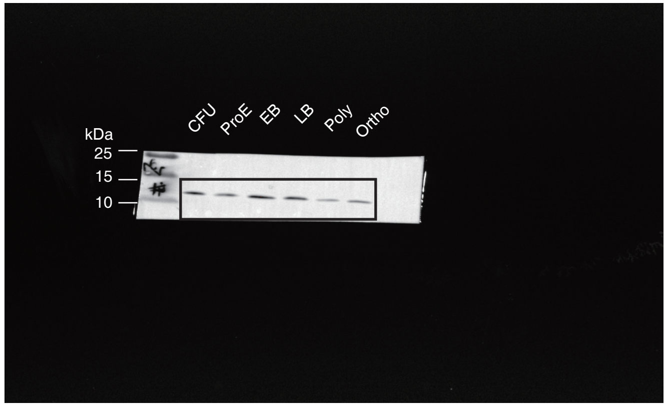

Histone H3

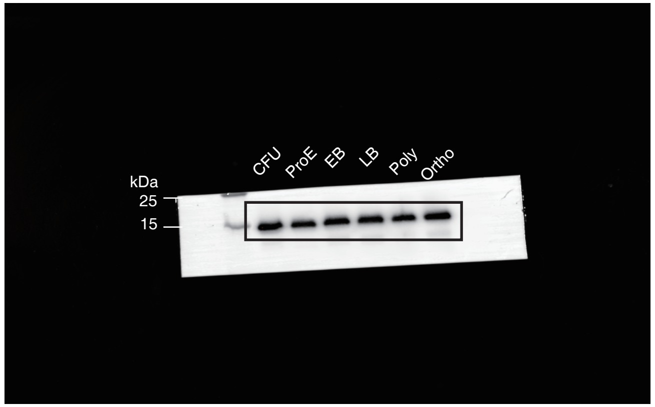

Figure S8. Gel images for Western blot analyses in Fig. 4A. Western blot analysis of different histone markers levels at different erythroid differentiation stages.

GAPDH

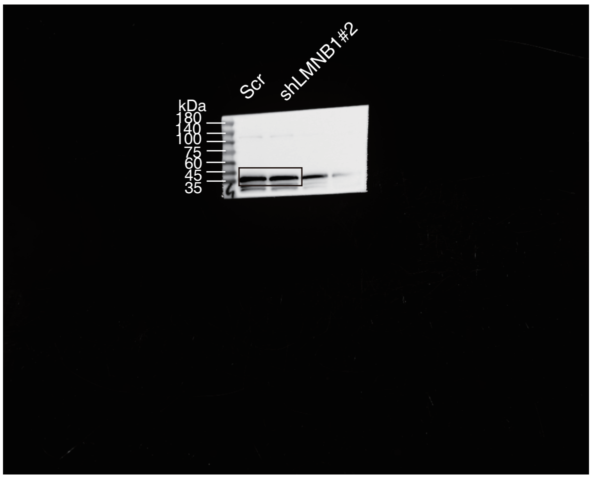

LaminB1

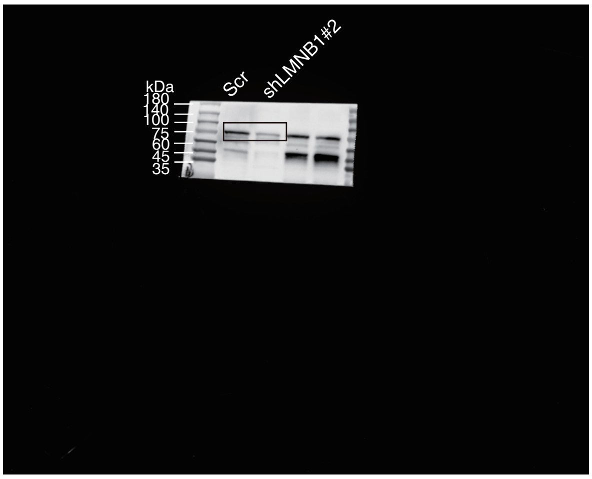

H3K9me3

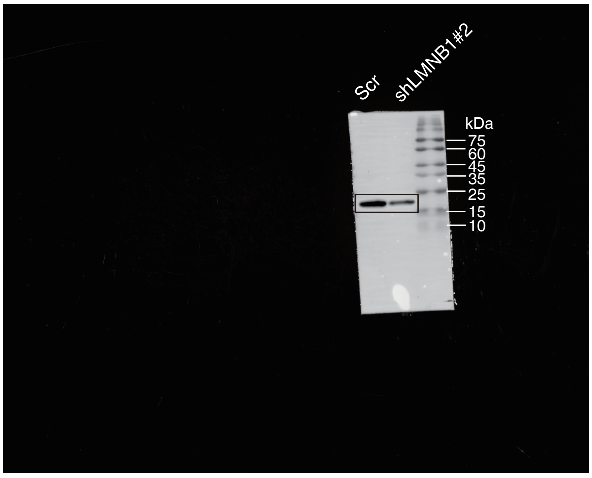

Figure S9. Gel images for Western blot analyses in Fig. S5C. Western blot analysis of H3K9me3 and Lamin B1 in control and LMNB1 knockdown group at day12 within infected at day7.
