# Supplementary material for: Dynamic changes in Lamin B1 and heterochromatin coincide with chromatin condensation during human erythropoiesis
Source: Genome Biol. 2026 Mar 24;27:148. doi: 10.1186/s13059-026-04025-x (PMC13134142; doi:10.1186/s13059-026-04025-x)
Supplement: Supplementary file 3 — Additional file 3: Table S2. Table of reagent/resource catalog number and software version. [file 13059_2026_4025_MOESM3_ESM.docx]

**Table of reagent/resource catalog number and software version.**

| **REAGENT or RESOURCE** | **SOURCE** | **IDENTIFIER** |
| --- | --- | --- |
| Antibodies | | |
| Lamin A/C | CST | #4777 |
| Lamin B1 | CST | #68591 |
| H3K4me1 | CST | #5326 |
| H3K9me2 | Abcam | #ab1220 |
| H3K9me3 | Abcam | #ab176916 |
| H3K27ac | Abcam | #ab4729 |
| H3 | CST | #4499 |
| H2AK119ub1 | CST | #8240 |
| H3K27me3 | CST | #9733 |
| H4K20me1 | abcam | #ab177188 |
| H4K20me3 | CST | #5737 |
| GPA | BD Biosciences | #555570 |
| CD36 | Biolegend | #336208 |
| CD71 | BD Biosciences | #555537 |
| CD105 | BD Biosciences | #562408 |
| XCp 19 Orange | Metasystems | #D-0319-100-OR |
| XCp 2 Green | Metasystems | #D-0302-100-F1 |
| Chemicals, peptides, and recombinant proteins | | |
| EPO | Stemcell | #78007 |
| SCF | Abclonal | #RP00124 |
| AB serum | Gemini | 100-512 |
| Hoechst33342 | beyotime | #C1022 |
| DRAQ5 | abcam | #Ab108410 |
| Insulin | TargetMol | #T8221 |
| Heperin | TargetMol | #T8221 |
| DEX | TargetMol | #T1076 |
| FBS | CORNING | #35-081-CV |
| IMDM | gibco | #12440-053 |
| SFEMII | Stemcell | #09655 |
| Critical commercial assays | | |
| Trueprep DNA library prep kit V2 for illuminated | Vazyme | TD501; TD503 |
| HiScript II Q Select RT SuperMix for qPCR (gDNA wiper) | Vazyme | R233-01 |
| EasySep™ Human CD34 Positive Selection Kit II | Stemcell | # 17856 |
| QIAGEN PCR Purification Kit | QIAGEN | #28106 |
| Giemsa staining kit | Solarbio | G4640 |
| Deposited data | | |
| Hi-C, ChIP-seq and RNA-seq data | This paper | https://ngdc.cncb.ac.cn/gsa-human/browse/HRA012152 |
| RNA-seq data from different stage during erythropiesis |  | GSE107218 |
| Experimental models: Cell lines | | |
| UCB-HSCs | Umbilical Cord Blood-Derived Hematopoietic Stem Cells | N/A |
| 293T cells | HCB lab | N/A |
| Oligonucleotides | | |
| *LMNA* Knock down#1:  5’-CGACTGGTGGAGATTGACAAT-3’ | This paper | N/A |
| *LMNA* Knock down#2:  5’- GAAGCAACTTCAGGATGAGAT -3’ | This paper | N/A |
| *LMNB1* Knock down#1:  5’- GGAGACACATCAGTCAGTTAT -3’ | This paper | N/A |
| *LMNB1* Knock down#2:  5’- GCATGAGAATTGAGAGCCTTT -3’ | This paper | N/A |
| *LMNA* Knock down-qPCR-F: ACGGCTCTCATCAACTCCACTG | This paper | N/A |
| *LMNA* Knock down-qPCR-R: TCCTCATCCTCGTCGTCCTCAA | This paper | N/A |
| *LMNB1* Knock down-qPCR-F: AAGCAGCTGGAGTGGTTGTT | This paper | N/A |
| *LMNB1* Knock down-qPCR-R: TTGGATGCTCTTGGGGTTC | This paper | N/A |
| Software and algorithms | | |
| CellProfiler (4.2.8) | [McQuin et al., 2018](#_ENREF_13) | https://cellprofiler.org |
| Bowtie2 (version 2.3.5.1) | [Langmead and Salzberg, 2012](#_ENREF_7) | http://bowtie-bio.sourceforge.net/bowtie2/index.shtml |
| MACS2 (v2.1.1.20160309) | [Yong et al., 2008](#_ENREF_24) | https://pypi.org/project/MACS2/ |
| ChIA-PET2 (0.9.3) | Guipeng Li et al., 2016 | https://github.com/GuipengLi/ChIA-PET2 |
| Hic-pro ( [v3.1.0](https://github.com/nservant/HiC-Pro/releases/tag/v3.1.0)) | Servant N et al., 2015 | [GitHub - nservant/HiC-Pro: HiC-Pro: An optimized and flexible pipeline for Hi-C data processing](https://github.com/nservant/HiC-Pro) |
| Hicexplorer (V3.7.2) | Joachim Wolff et al., 2022 | [GitHub - deeptools/HiCExplorer: HiCExplorer is a powerful and easy to use set of tools to process, normalize and visualize Hi-C data.](https://github.com/deeptools/HiCExplorer) |
| Chrom3D ([v1.0.2](https://github.com/Chrom3D/Chrom3D/releases/tag/v1.0.2)） | Jonas Paulsen et al., 2017 | [GitHub - Chrom3D/Chrom3D](https://github.com/Chrom3D/Chrom3D) |
| STAR (2.7.11b) | [Alexander Dobin](https://pubmed.ncbi.nlm.nih.gov/?term=Dobin+A&cauthor_id=23104886) et al., 2013 | [GitHub - alexdobin/STAR: RNA-seq aligner](https://github.com/alexdobin/STAR) |
| EDD (v1.1.0) | [Eivind Lund](https://pubmed.ncbi.nlm.nih.gov/?term=Lund+E&cauthor_id=24782521) et al., 2014 | [GitHub - CollasLab/edd: Enriched Domain Detector for ChIP-seq data](https://github.com/CollasLab/edd) |
| RSEM (v1.3.3） | [Bo Li](http://bli25ucb.github.io/) , et al., 2011 | [GitHub - deweylab/RSEM: RSEM: accurate quantification of gene and isoform expression from RNA-Seq data](https://github.com/deweylab/RSEM) |
| Fastp (v0.26.0) | Shifu Chen, et al., 2023 | [GitHub - OpenGene/fastp: An ultra-fast all-in-one FASTQ preprocessor (QC/adapters/trimming/filtering/splitting/merging...)](https://github.com/OpenGene/fastp) |
| Homer (v5.1) | Heinz et al, et al., 2010 | [GitHub - sipcapture/homer: HOMER - 100% Open-Source SIP, VoIP, RTC Packet Capture & Monitoring](https://github.com/sipcapture/homer) |
| Juicer (v1.6) | Neva C. Durand, et al., 2016 | [GitHub - aidenlab/juicer: A One-Click System for Analyzing Loop-Resolution Hi-C Experiments](https://github.com/aidenlab/juicer) |
